# Ruminal microbiota-host crosstalks promote ruminal epithelial development in neonatal lambs with alfalfa hay introduction

**DOI:** 10.1128/msystems.01034-23

**Published:** 2024-01-05

**Authors:** Gaorui Bian, Shiqiang Yu, Chao Cheng, Haixuan Huang, Junhua Liu

**Affiliations:** 1 College of Animal Science and Food Engineering, Jinling Institute of Technology, Nanjing, China; 2 Laboratory of Gastrointestinal Microbiology, Jiangsu Key Laboratory of Gastrointestinal Nutrition and Animal Health, College of Animal Science and Technology, Nanjing Agricultural University, Nanjing, China; 3 National Center for International Research on Animal Gut Nutrition, Nanjing Agricultural University, Nanjing, China; 4 College of Food Science and Technology, Nanjing Agricultural University, Nanjing, China; State Key Laboratory of Mycology, Institute of Microbiology, Chinese Academy of Sciences, Beijing, China

**Keywords:** sucking lambs, alfalfa hay, rumen development, microbial community, epithelial function

## Abstract

Ruminal microbiota is gradually established after birth, while microbiota maturation could be highly diverse because of varied solid dietary accessibility. However, how the ruminal microbiota accreted from postnatal hay diets alters rumen epithelial development, and how this affects animal health remains largely unknown. Here, neonatal lambs were introduced to starchy corn-soybean starter or corn-soybean starter + alfalfa hay (AH) to investigate the influences of early life ruminal microbiome on rumen epithelial development using integrated 16s rRNA sequencing-metagenome-transcriptome approaches. The results showed that AH introduction elevated average daily weight gain, rumen weight and volume, rumen epithelial papillae length, and rumen muscle layer thickness. Meanwhile, the relative abundance of fibrolytic bacteria (*Christensenellaceae* R-7 group, *Prevotellaceae* UCG-001, and *Succinivibrio*), acetate producer (*Acetitomaculum* and *Mitsuokella),* and propionate producer *Succiniclasticum* was increased in the rumen content by AH supplementation (*P* < 0.05). Moreover, AH introduction decreased the relative abundance of total CAZymes, CBM, and GH and increased the abundance of KO genes related to volatile fatty acid (VFA) generation in the rumen content. AH lambs had a higher relative abundance of *Succiniclasticum*, *Megasphaera, Succinivibrio*, and *Suttonella* (*P* < 0.05), while a lower relative abundance of *Cloacibacillus*, *Desulfovibrio*, *Dialister*, *Intestinimonas*, *Parabacteroides*, and *Pseudoscardovia* (*P* < 0.05) in the rumen epithelial samples. Furthermore, these alterations in ruminal microbial structure and function resulted in ruminal epithelial cell proliferation and development pathways activation. In summary, AH introduction benefited ruminal fiber degradation and VFA generation bacteria colonization and promoted ruminal epithelial development. These findings provide new insights into ruminal microbial–host interactions in the early life.

**IMPORTANCE:**

While it is established that a fiber-rich diet promotes rumen development in lambs, further research is needed to investigate the precise response of rumen microbiota and epithelium to high-quality alfalfa hay. Here, we observed that the inclusion of alfalfa hay led to a discernible alteration in the developmental trajectory of the rumen. Notably, there was a favorable shift in the rumen's volume, morphology, and the development of rumen papillae. Furthermore, ruminal microbial structure and function resulted in ruminal epithelial cell proliferation and development pathways activation, collectively provide compelling evidence supporting the capacity of alfalfa hay to enhance rumen development and health through ruminal micrbiota-host crosstalks. Our findings elucidate the functional response of the rumen to alfalfa hay introduction, providing new insights into strategies for promoting healthy development of the rumen in young ruminants.

## INTRODUCTION

Gastrointestinal microorganisms exert fundamental roles in host development and health (1, 2). Diet is the strongest modulator governing gastrointestinal microbial ecology via nutrient provision. The myriad of metabolites derived from the microbial conversion of diet nutrients exert their effects within the host as signaling molecules and substrates for metabolic reactions (3). Therefore, diet-microbe-host have formed a complex symbiont during long time co-evolution history. However, the study of nutrients (plant components)-microbe-host (animal) interactions remains great challenging due to the high degree of crosstalk both within and across kingdoms (4, 5).

The first part of multichambered stomach-rumen represents a highly typical and natural fermentation chamber inhabited by complicated and diverse microbes, including bacteria, archaea, fungi, and ciliated protozoa (6). This arrangement enables ruminants to efficiently convert plant materials into precursors (volatile fatty acids [VFAs], microbial protein, and vitamins) of high-value meat and milk (7). Within this symbiotic system, the ruminal microbes provide energy and nutrients from plant components to the host animal, and the host rumen gives a suitable survival habitat and metabolism environment for microorganisms (8). In response to this complex microbial ecosystem, rumen tissue has evolved a series of distinct anatomical features, including ruminal keratinized papillae formation and muscularization (9). Rumen stratified squamous epithelium provides several physiologically vital functions, including absorption, metabolism, and protection (10). Rumen muscular contractions mix fresh feed with microorganisms and soak the epithelium with fermentation fluids so the microbial VFAs can be absorbed and finally affecting rumen motions and rumination (11). Hence, the optimal rumen epithelial and muscular development plays crucial and central roles in ruminant health and performance.

Ruminant animals and ruminal microorganisms have a symbiotic relationship that facilitates fiber degradation in long time co-evolution history, but domestic ruminants in the current intensive production system are often supplied with a high-grain diet and little fiber. Alfalfa, known as “king of pasture,” is the main high-quality source of pasture for domestic ruminants. Starchy corn-soybean is a typical high-grain diet commonly fed to ruminants to improve animal performance and economic benefits of ruminant agriculture. Ruminant nutritionist indicates that these two typical solid diet introductions exert their own unique effects on the development of ruminal epithelial papillae or musculature (12, 13). In the early life, differentially altered rumen microbial structure and the production of VFAs by early nutritional intervention have been widely recognized as critical factors that drive rumen epithelial development (14). Thus, the highly dynamic pioneer ruminal microbiome before the establishment of a stable adult microbiome is the window of opportunity for rumen development modulations. Here, neonatal lambs were introduced to starchy corn-soybean starter or corn-soybean starter +alfalfa hay to investigate the influences of the early life ruminal microbiome on rumen epithelial development using integrated 16s rRNA sequencing-metagenome-transcriptome approaches.

## MATERIALS AND METHODS

### Experimental animals and feeding management

This animal experiment was conducted in accordance with the guidelines of the Animal Care and Use Committee at Nanjing Agricultural University (SYXK2017-0027). Twelve 14-day-old healthy sucking twin lambs, with similar initial body weights, were selected from a flock of twin lambs born within a week. The lambs were randomly divided into two groups to receive different feed strategies: a group supplemented with milk + starchy corn-soybean starter (CON; *n* = 6) and a group supplemented with milk + corn-soybean starter + alfalfa hay (AH; *n* = 6). Furthermore, to eliminate the influence of genetic factors, we assigned the two lambs from the same twin pair to the CON group and the AH group, respectively. All lambs were fed 600 mL/d milk equaling 10% of their initial average body weight and had free access to solid feed. The mixed goat milk powder was provided four times daily (07:00, 12:00, 17:00, and 22:00), and the solid diets were provided twice daily (08:00 and 17:00). The nutritional levels of alfalfa hay and corn-soybean starter are shown in Table S1.

### Sample collection

At 42 days of age, after a 12-hour fasting period, the lambs were weighed and slaughtered by exsanguination. The rumen was immediately removed and weighed. The rumen contents were collected and thoroughly mixed. A subsample of 150 mL rumen fluid was filtered through four layers of cheesecloth and stored at −20°C for VFA analysis. Another subsample of 10 g rumen contents was stored at −80℃ for microbial DNA extraction and subsequent metagenomics sequencing. The rumen was then emptied, rinsed with water, blotted dry, and weighed again to obtain the emptied rumen weight. The rumen volume was calculated as the difference between the rumen wet weight and dry weight divided by the density of water (1 g/mL). Three 3 × 3 cm sections of the rumen wall sample were collected from the ventral sac for rumen papilla morphology measurement or rumen epithelial morphology analysis. Another section of the rumen wall sample (3 × 3 cm) from the ventral sac was scraped gently with a sterile glass slide to obtain the rumen epithelium. The scraped epithelium was stored in liquid nitrogen for tissue RNA and epithelial microbial DNA extraction.

### Determination of fermentation parameters

The rumen content was filtered using four layers of cheesecloth, and the pH was promptly measured using a digital pH meter (PHS-3C; Shanghai Yueping Scientific Instrument Co., Ltd., Shanghai, China). The VFA concentrations in rumen fluid samples were analyzed using a gas chromatograph (GC-7890B; Agilent Technologies, Santa Clara, CA) equipped with a capillary column (30 m × 0.25 mm × 0.25 mm; DB-FFAP; Agilent Technologies) and a flame ionization detector. The relative correction factor of organic acids can be calculated based on the peak area of the standard sample and the internal standard. The concentration and proportion of VFAs in each sample can be calculated based on the concentration of VFAs and the ratio of their peak areas.

### Histological measurements

The rumen papilla morphology and light microscopy histomorphometric analysis were detected through the method described by Sha et al. (15). Rumen papilla density was determined by counting the number of papillae in a 1 × 1 cm area of the rumen sac wall. The length and width of each selected papilla were measured using a caliper, and the absorption area of the rumen epithelium was calculated as papilla length × width × density × 2. Another part of the rumen tissue was embedded in paraffin and sectioned (H&E staining). The thickness of the rumen epithelial layer and muscular layer was measured using a Nikon upright microscope [Nikon Precision (Shanghai) Co., Ltd., Shanghai, China] and Image-Pro Plus 6.0 software.

### Microbial DNA extraction

Microorganism genomic DNA from rumen contents and rumen epithelium was extracted using DNA extraction kits (E.Z.N.A. soil DNA kit; Omega Bio-Tek, USA) following the manufacturer’s recommendations. The quantity and quality of DNA samples were assessed using an ND-1000 spectrophotometer (NanoDrop, Wilmington, DE, USA) and checked on a 1% agarose gel. The DNA samples were then stored at −80℃ for further sequencing analysis.

### 16S rRNA gene and metagenomic sequencing

For the microbial analysis of rumen contents and rumen epithelium, we amplified the V3-V4 region of the 16S rRNA gene using universal primers (341F [5’- CCTAYGGGRBGCASCAG-3′] and 806R [5′-GGACTACNNGGGTATCTAAT-3′]). The resulting amplicons were purified using the QIAquick PCR purification kit (Qiagen, Hilden, Germany), and then library construction was performed following the Illumina manufacturer’s instructions. All libraries were sequenced using the Illumina MiSeq PE-250 platform.

The DNA samples from rumen contents underwent additional shotgun metagenomic sequencing. The genomic DNA was fragmented to an average size of 400 bp using a Covaris M220 (Gene Company Limited, China). For paired-end library construction, the NEXTFLEX Rapid DNA-Seq Kit (Bioo Scientific, Austin, TX, USA) was employed. The pooled libraries were then sequenced using an Illumina HiSeq X Ten platform with 2 × 150 bp sequencing.

### 16S rRNA analysis

After the paired-end sequencing of the qualified amplicon libraries on the Illumina NovaSeq PE250 platform, we employed FLASH (http://ccb.jhu.edu/software/FLASH/) for read splicing and assembly. Fastqc (https://www.bioinformatics.babraham.ac.uk/projects/fastqc/) was utilized for quality filtering, and UCHIME (http://www.drive5.com/usearch/manual/uchime_algo.html) was applied for the detection and removal of chimeras. The available plugins within QIIME2 were utilized for taxonomic analysis. Specifically, the DADA2 plugin was employed to inspect the sequence quality and denoise the reads, resulting in the determination of the abundance table of amplicon sequencing variants (ASVs). The classify-sklearn plugin was used to train the feature classifier, and the naïve Bayesian taxonomic classifier was used to annotate the taxonomy of the ASVs.

### Metagenomic analysis

In order to obtain high-quality clean reads, sequences with over 50% bases having quality scores below 20, or reads with more than 10% unidentified nucleotides were filtered out. Additionally, reads contaminated by adaptors and sheep host sequences were eliminated using Bowtie2 (16). The sequence data were assembled using MEGAHIT (17). K-mers ranging from 21 to 99 were generated for sample-derived assembly, and the unmapped reads of each sample were pooled for re-assembly using MEGAHIT to generate a mixed assembly. Afterward, the overall *de novo* assembly statistics were assessed by realigning singleton reads using BWA (18). MetaGeneMark (19) was used to predict contigs (>500 bp) from each sample, and the Open Reading Frames (ORFs) were obtained from the assembled contigs. Then, CD-HIT (20) was applied for clustering, resulting in a non-redundant dataset with a sequence identify cutoff of 95% identity and read coverage of 90%. Next, the obtained reads were mapped to the non-redundant gene dataset, and SOAPaligner (21) was used to calculate the gene abundance with a similarity of 95%. All ORFs were annotated to the CAZY functional databases using DIAMOND (22).

### Rumen RNA extraction and processing of transcriptome sequencing

We used the Trizol reagent kit (Invitrogen, Carlsbad, USA) to extract total RNA from the rumen epithelial samples following the manufacturer’s instructions. RNA quality was assessed using an Agilent 2100 Bioanalyzer (Agilent Technologies, Palo Alto, CA, USA) and non-RNA degrading agarose gel electrophoresis. Samples with an RNA integrity number greater than 7.0 were selected for sequencing.

Each of the 12 sequencing libraries was prepared following the Illumina TruSeq^T^ RNA sample preparation protocol. The paired-end sequencing (2 × 125 bp) was performed using the Illumina HiSeq 2500 platform. Fastp (version 0.18.0) was used to remove reads containing adapters and reads with more than 10% unknown nucleotides (N), as well as to delete reads with more than 50% low-quality bases (*Q*-value ≤20). The index of the reference genome was built using Bowtie2 (version 2.2.8) (16), and the clean sequences were aligned to the sheep genome (Ovis aries, Ensembl_release 96) using HISAT 2.2.4. StringTie software was used to calculate the fragments per kilobase of transcript per million mapped reads (FPKM), quantifying the gene expression abundance and variations. The DESeq2 software was used to filter differentially expressed genes (DEGs) between the CON group and the AH group, considering genes with a false discovery rate (FDR) less than 0.05 and an absolute fold change ≥2 as DEGs. The DEGs were subjected to pathway enrichment analysis using the Kyoto Encyclopedia of Genes and Genomes (KEGG). The enrichment analysis of DEGs based on Gene Ontology (GO) was conducted using DAVID (version 6.8) (23).

Weighted gene co-expression network analysis (WGCNA) was conducted to elucidate the correlation between the host transcriptome and rumen development indices. For this analysis, all expressed genes (38,075 in total, with FPKM > 0.5 in at least one sample) from rumen tissue samples collected from all lambs were included. The analysis was performed using R Studio (version 1.3.1335). Following the application of soft thresholding power, we constructed a signed network based on Pearson correlation. Subsequently, we conducted a Pearson correlation analysis between gene expression and the rumen development indices for each module, which represents clusters of highly interconnected genes. We conducted module detection (blockwise modules in WGCNA) using the following parameters: a maximum module size of 12,000 genes, a minimum module size of 100 genes, and a reassignment threshold of 0.25.

### Statistical analyses

The taxonomy data from the experiment were initially organized in Excel and then statistically analyzed using the SPSS software package version 25 (IBM Corp., Armonk, NY, USA). The normality of the distribution of variables was tested using the Shapiro–Wilk test. When the variable distributions were assumed normal, the independent samples *t*-test procedure was used; otherwise, the Kruskal–Wallis procedure was used. When *P* ≤ 0.05, it is considered as a significant difference, and when 0.05 < *P* < 0.01, it is considered as having a trend of change.

## RESULTS

### Animal performance, rumen morphology, and rumen fermentation

As shown in Table 1, compared to the CON group, AH supplementation significantly increased body weight gain (*P* = 0.002) and average daily weight gain (*P* = 0.002) of lambs. Meanwhile, compared to the CON group, the lambs in the AH group had larger rumen wet weight (*P* = 0.002), emptied rumen weight (*P* = 0.009), emptied rumen weight/body weight ratio (*P* = 0.047), and rumen volume (Table 1; Fig. S1A through D). Moreover, the rumen epithelial papilla length (*P* = 0.011) and muscular layer thickness (*P* = 0.003) were significantly increased by AH supplementation (Table 1; Fig. S1G and H).

**TABLE 1:** Animal performance and rumen epithelial morphology of lambs

Item	CON^*a*^	AH	*P-*value
Animal performance			
Initial body weight, kg	5.85 ± 0.752	5.78 ± 1.084	0.904
Final body weight, kg	9.53 ± 0.532	11.48 ± 0.474	0.059
Weight gain, kg	3.68 ± 0.380	5.70 ± 0.310	0.002
Average daily weight gain, g	136.11 ± 14.059	211.11 ± 11.484	0.002
Rumen organ index and epithelial morphology			
Rumen wet weight, g	679.67 ± 82.552	1,080.67 ± 42.879	0.002
Emptied rumen weight, g	170.00 ± 11.054	238.83 ± 18.113	0.009
Rumen volume, mL	1,077.50 ± 106.768	1,871.43 ± 59.121	≤0.001
Emptied rumen weight/body weight, %	1.79 ± 0.061	2.09 ± 0.118	0.047
Papilla length, mm	2.52 ± 0.127	3.43 ± 0.262	0.011
Papilla width, mm	1.25 ± 0.091	1.50 ± 0.073	0.063
Papilla density, cm^2^	159.83 ± 18.060	154.66 ± 20.440	0.882
Epithelial absorption area, mm^2^/cm^2^	2,027.11 ± 286.021	3,168.79 ± 476.348	0.067
Muscle layer thickness, μm	864.83 ± 52.856	1,118.22 ± 36.894	0.003

^*a*^ The values shown are means ± SEM (standard error of the mean); *P* < 0.05 indicated that mean values were significantly different. CON: control group, AH: alfalfa hay group.

The results of rumen fermentation parameters in lambs are shown in Table 2. Compared to the CON group, the AH group had higher rumen fluid pH (*P* = 0.001) and lower total VFA level (*P* = 0.031). Furthermore, there was an increasing trend in the proportion of propionate (*P* = 0.076) and a decreasing trend in the proportion of butyrate (*P* = 0.052).

**TABLE 2:** The ruminal fermentation parameters of lambs^*a*^

Item	CON	AH	*P*-value
pH	5.14 ± 0.052	5.70 ± 0.104	0.001
Total VFA, mM	172.32 ± 9.853	135.38 ± 11.012	0.031
Acetate, %	50.55 ± 6.284	53.17 ± 2.707	0.269
Propionate, %	24.47 ± 2.641	33.70 ± 3.843	0.076
Isobutyrate, %	0.29 ± 0.036	0.30 ± 0.060	0.839
Butyrate, %	20.93 ± 5.100	9.38 ± 1.215	0.052
Isovalerate, %	0.32 ± 0.047	0.42 ± 0.056	0.173
Valerate, %	3.44 ± 0.385	3.03 ± 0.454	0.501
Acetate/Propionate	2.26 ± 0.432	1.69 ± 0.216	0.269

^*a*^ The values shown are means ± SEM (standard error of the mean); *P* < 0.05 indicated that mean values were significantly different. CON: control group, AH: alfalfa hay group.

### Composition and function of microbial community in the rumen contents of lambs

**Fig 1:**
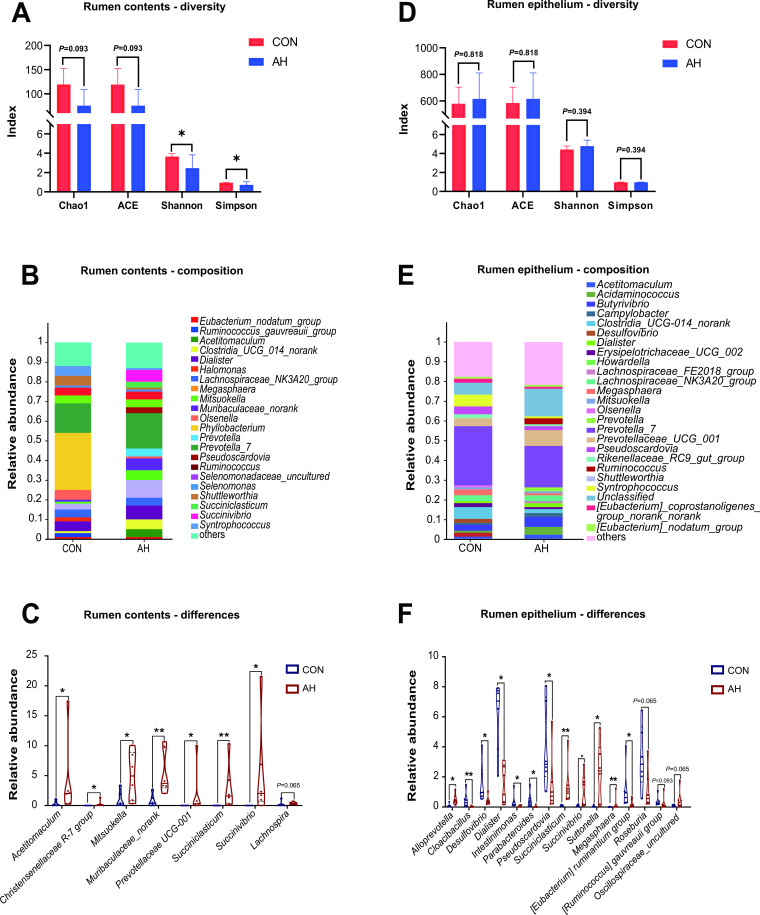
Comparative analysis of microbial diversity (**A and D**), genus-level composition (**B and E**), and different genus (**C and F**) between CON and AH group in rumen contents (**A, B, and C**) and epithelium (**D, E, and F**). CON: control group, AH: alfalfa hay group.

As shown in Fig. S2A, the rarefaction curves of all samples tended to flatten, indicating that the sequencing depth was sufficient to cover most bacteria. PCoA analysis indicated a significant difference between the rumen microbial populations of the two groups in Fig. S2B (*P* = 0.004). Compared to the CON group, AH introduction significantly decreased the Shannon and Simpson indices of rumen microbial communities (*P* < 0.05), and Chao1 and ACE also showed a decreasing trend in Fig. 1A (*P* = 0.093).

As shown in Fig. S3A, a total of 24 phyla were detected in the rumen contents samples, and Firmicutes, Bacteroidota, Proteobacteria, Actinobacteriota, and Euryarchaeota were the main phylum among all lambs. We analyzed the major bacterial phyla at the level of rumen contents and found no significant differences between the two groups (Fig. S4A). A total of 363 genera were detected, of which 44 had a relative abundance greater than 1% in at least one sample (Fig. 1B). Subsequently, we conducted further analysis at the bacterial genus level. In the PCoA plot, a distinct separation was observed between the two groups (Fig. S4B). Moreover, AH supplementation increased the relative abundance of *Muribaculaceae_norank*, *Succiniclasticum*, *Acetitomaculum*, *Christensenellaceae* R-7 group, *Mitsuokella*, *Prevotellaceae* UCG-001, and *Succinivibrio* (*P* < 0.05) (Fig. 1C).

**Fig 2:**
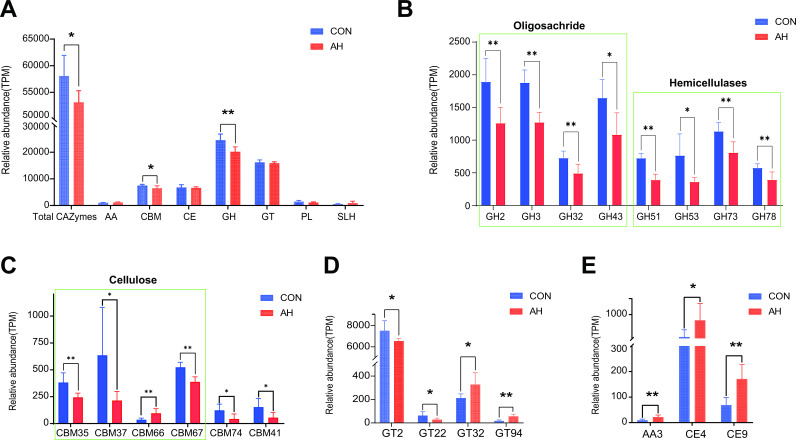
Differential expression of microbial CAZymes at different levels in rumen contents. (A) Class level; (B) GH family; (C) CBM family; (D) GT family; (E) Other families. *Indicates a significant difference between the two groups (*P* < 0.05); **Indicates an extremely significant difference between the two groups (*P* < 0.01). CON: control group, AH: alfalfa hay group.

Rumen microbial secretion of various enzymes can degrade carbohydrates. We focused on the differences in ruminal bacterial CAZyme profiles between CON and AH groups. Clear separation of CAZymes between the two groups was observed at the class (Fig. S5A) and family (Fig. S5B) levels. Among the CAZymes involved in carbohydrate degradation (including cellulose, hemicellulose, starch, and lignin), AH introduction decreased the relative abundance of total CAZymes, carbohydrate-binding modules(CBM), and glycoside hydrolases (GH) at the class level (*P* < 0.05). Furthermore, in the comparison at the family level, we found that in the CON group, eight GH families (GH2, GH3, GH32, GH43, GH51, GH53, GH73, GH78), five CBM families (CBM35, CBM37, CBM41, CBM67, CBM74), and two glycosyl transferases (GT) families (GT2, GT22) were significantly enriched (*P* < 0.05). CBM66, GT32, GT94, AA3 (auxiliary activities [AA]), CE4 (carbohydrate esterases(CE), and CE9 were significantly enriched in the AH group (*P* < 0.05) (Fig. 2A through E). These altered carbohydrate enzymes are mainly related to the degradation of oligosaccharides, hemicelluloses, and cellulose.

**Fig 3:**
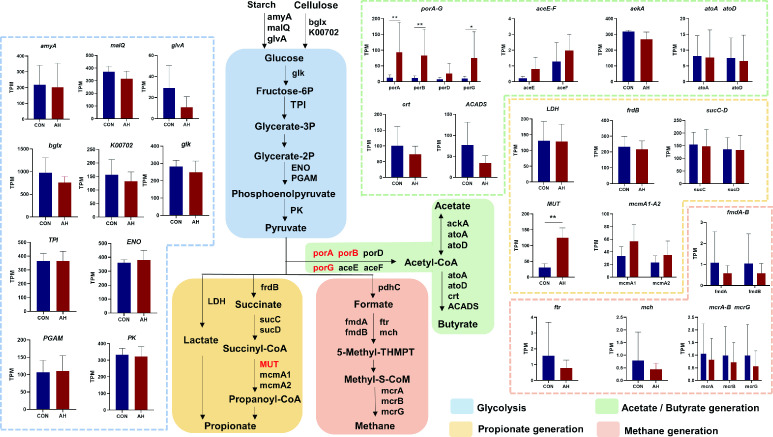
Glycolysis and the metabolism pathway of VFAs in the rumen. *Indicates a significant difference between the two groups (*P* < 0.05); **Indicates an extremely significant difference between the two groups (*P* < 0.01). CON: control group, AH: alfalfa hay group.

Figure 3 depicts the pathways of acetate, propionate, butyrate, and methane generation involving numerous enzyme-encoding genes during starch and cellulose digestion. Metagenomic sequencing results revealed changes in the abundance of KO genes related to fermentation pathways between the two groups. In the AH group, the abundance of porA, porB, and porG involved in the acetate and butyrate generation pathway increased significantly (*P* < 0.05), and the abundance of MUT associated with the propionate generation pathway increased significantly (*P* < 0.05). This suggests that the early introduction of AH promotes VFA generation in the rumen of suckling lambs.

### Characterization of bacterial community attached to rumen epithelia of lambs

As shown in Fig. S2C, the rarefaction curves of lamb rumen epithelial bacteria tended to flatten out, indicating that the sequencing depth and data volume were sufficient to cover the microbial community. The results of PCoA analysis showed that the rumen epithelial bacteria between the two groups could be separated (*P* < 0.05; Fig. S2D). In Fig. 1D, the results of alpha diversity analysis of the rumen epithelial microbiota between the two groups of lambs are presented. There was no significant difference in Chao1, ACE, Shannon, and Simpson indices between the two groups.

As shown in Fig. S3B, a total of 33 phyla were detected in the rumen epithelial samples. After screening with a relative abundance >1% in at least one sample, a total of eight phyla were screened out, including Acidobacteriota, Synergistota, Desulfobacterota, Campylobacterota, Actinobacteriota, Proteobacteria, Bacteroidota, and Firmicutes. The dominant phyla in both groups were Firmicutes and Bacteroidota. Figure 1E shows the sequencing results at the genus level for the rumen epithelial bacteria in both groups of lambs, with a total of 688 genera detected, and 54 genera with a relative abundance greater than 1% in at least one group. Further analysis at the phylum and genus levels of epithelial microbiota revealed clear separation in the PCoA results for both (Fig. S6A and B). Compared to the CON group, the relative abundance of Actinobacteriota was significantly decreased (*P* = 0.022), while the relative abundance of Proteobacteria was significantly increased (*P* = 0.011) in the AH group (Fig. S7).

Figure 1F shows the differential genus-level microbiota between the two groups. Compared to the CON group, AH lambs had the higher relative abundance of *Succiniclasticum*, *Megasphaera, Alloprevotella*, *Succinivibrio*, and *Suttonella* (*P* < 0.05), while lower relative abundance of *Cloacibacillus*, *Desulfovibrio*, *Dialister*, *Intestinimonas*, *Parabacteroides*, *Pseudoscardovia*, and [*Eubacterium*] *ruminantium grou*p (*P* < 0.05) in the rumen epithelial samples. The relative abundance of *Roseburia* (*P* = 0.065) and [*Ruminococcus*] *gauvreauii group* (*P* = 0.093) showed a decreasing trend, and the relative abundance of *Oscillospiraceae_uncultured* showed an increasing trend (*P* = 0.065) in rumen epithelia of AH group.

### Rumen bacterial co-occurrence patterns between rumen contents and epithelia

**Fig 4:**
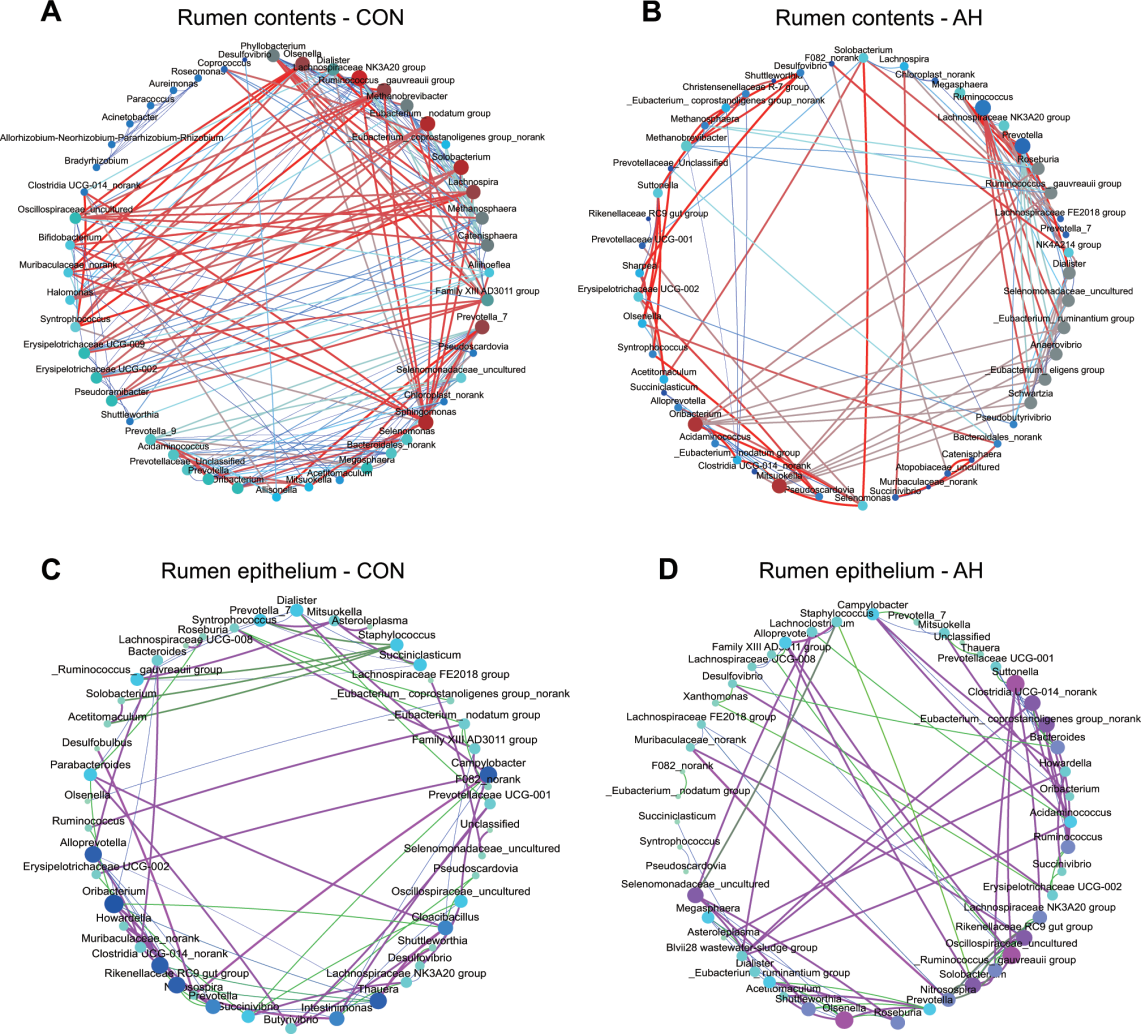
Microbial network correlation between the CON group (**A and C**) and the AH group (**B and D**) in rumen contents (**A and B**) and epithelium (**C and D**). CON: control group, AH: alfalfa hay group.

In order to gain deeper insights into the microbial functional responses to AH introduction, we conducted an analysis of the correlated networks between the bacteria of the rumen contents and that attached to the rumen epithelium (Fig. 4A through D). We conducted network analysis for the top 50 genera within the bacterial community (|R| > 0.5, *P* < 0.05). In the rumen contents, we observed that the connectivity of sample degrees in the bacterial network was 266 for the AH group, whereas it was 460 for the CON group. This disparity suggests a reduction in the bacterial network complexity within the AH group. In the rumen epithelium, the connectivity of sample degrees in the bacterial network was 200 for the AH group, whereas it was 172 for the CON group, indicating an augmentation in the complexity of the bacterial network within the AH group. Additional detailed information regarding the network can be found in Table S2. These findings imply that the introduction of alfalfa hay induces a transition in the complexity of the rumen microbiota from the contents to the epithelia, potentially fostering rumen development in lambs.

### Transcriptional profiles of rumen epithelia

**Fig 5:**
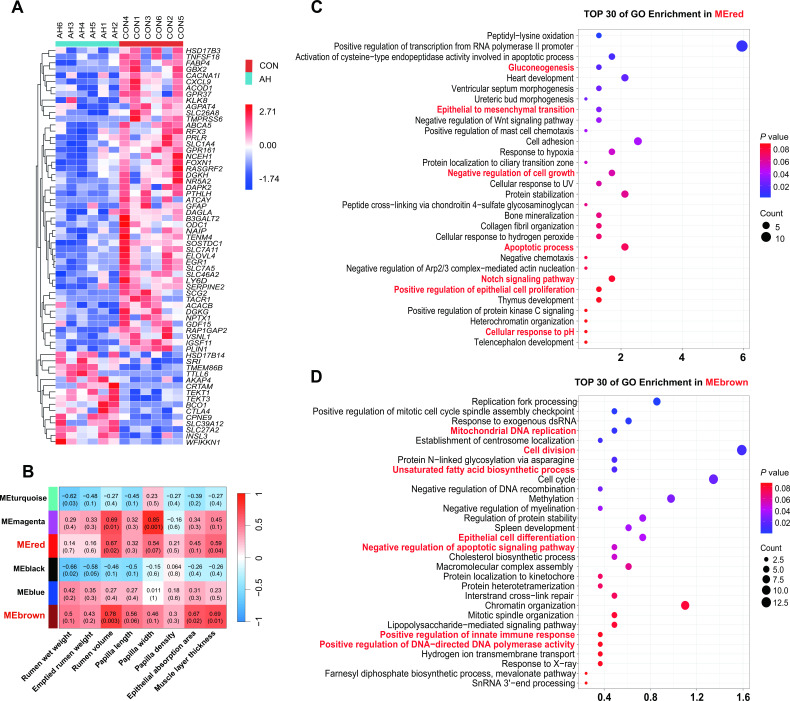
Analysis of rumen epithelial DEGs and WGCNA enrichment. (A) Major differential genes related to cell development, growth, and communication. (B) WGCNA of the correlation of host transcriptome with the rumen development indexes. (C) Top 30 GO pathway of genes significantly in the MEred module. (D) Top 30 GO pathway of genes significantly in the MEbrown module. CON: control group, AH: alfalfa hay group.

Transcriptome sequencing was applied to investigate the DEGs in rumen epithelium upon CON and AH group. There were 38,074 common genes between the two groups. Compared to the CON group, a total of 282 DEGs (FDR < 0.05; |log2(FC)| >2) in the AH group, including 204 significantly downregulated genes and 78 significantly upregulated genes (Fig. S8B). Furthermore, the PCoA plots were distinctly separated into two groups, indicating that different dietary interventions have altered gene expression in the rumen epithelia (Fig. S8A). Figure 5A shows the differences of major epithelial genes, these displayed genes were mainly related to cell development, growth, and communication. To further investigate the functions of DEGs, we performed KEGG and GO functional classification on genes exhibiting differences between the CON and AH groups. It was observed that genes significantly downregulated in the KEGG pathway were primarily enriched in Metabolism and Organismal Systems, while upregulated genes did not show significant enrichment (Fig. S9A). We performed an analysis leveraging DEGs, which led to the significant enrichment of 111 metabolic pathways within the biological process module. These substantially modified pathways were primarily linked to processes pertinent to “Cellular Transport, Metabolic Processes, Cellular and Biological Processes, and Signaling” (Fig. S8). Figure S9B provides a visual representation of the top 30 pathways within the Biological Process module.

Utilizing WGCNA, we grouped all expressed genes in the rumen epithelia into eight distinct gene modules. We observed that MEred (224 genes) and MEbrown (404 genes) exhibited positive correlations with rumen development indicators, as illustrated in Fig. 5B. Detailed information regarding the BP associated with MEred and MEbrown can be found in Table S3. The MEred module was linked to a range of metabolic pathways, encompassing gluconeogenesis, epithelial to mesenchymal transition, negative regulation of cell growth, apoptotic processes, the Notch signaling pathway, positive regulation of epithelial cell proliferation, and cellular responses to pH, as depicted in Fig. 5C. The MEbrown module is primarily enriched in mitochondrial DNA replication, cell division, unsaturated fatty acid biosynthetic process, epithelial cell differentiation, negative regulation of apoptotic signaling pathway, positive regulation of innate immune response, and positive regulation of DNA-directed DNA polymerase activity (Fig. 5D).

## DISCUSSION

The constituents of the diet represent a primary factor influencing the patterns of rumen development, which, in turn, consistently exert an impact on the health and performance of ruminants (24, 25). The dietary fiber in early starter diets has been identified as a key determinant influencing rumen development in lambs (12, 26). A fiber-rich diet is poised to exert a robust influence on the physiological aspects of rumen development, imparting essential physical stimuli necessary for its proper maturation (27). In our investigation, the fiber-rich alfalfa hay introduction facilitated rumen peristalsis, thereby expediting rumen microbial fermentation and augmenting rumen tissue development. The observed elevation in muscle layer thickness can be ascribed to the substantial crude fiber content found in alfalfa hay, which encouraged rumen peristalsis and rumination in lambs. Consequently, this accelerates the development of the rumen’s muscular layer, thereby enhancing its adaptability in terms of thickness and further augmenting the lamb’s digestive capabilities (28). Moreover, the incorporation of alfalfa hay, rich in dietary fiber, serves as an effective stimulus for the development of the rumen’s muscular layer through friction (29). Consequently, this leads to an augmentation in rumen volume and an increase in rumen weight. These adaptive alterations in rumen volume promote more efficient solid feed intake, ultimately fostering accelerated animal growth (30). Moreover, the augmentation in the length, width, and absorptive surface area of the ruminal epithelial papillae resulted in an expanded contact interface between the rumen epithelia and feed, consequently enhancing the efficiency of nutrient absorption. This discovery aligns with the findings of Zhuang et al. (31), who illustrated that supplementing solid feed in the diets of young ruminants amplifies rumen fermentation and fosters the proliferation of rumen papillae. VFAs in the rumen contribute to over 70% of the body’s energy, and significant alterations in these acids can have a profound impact on overall development and nutrient absorption. The addition of alfalfa hay to the lamb’s diet leads to a notable reduction in the total VFA content within the rumen, along with a declining trend in the proportion of butyric acid. These findings implied that the produced VFAs may be efficiently absorbed by the extended epithelial surface area, subsequently converted into energy for the lamb’s metabolic consumption (32). In general, alfalfa hay introduction made the growth and development of lambs better, which benefited from the optimization of rumen physiological state and the rapid development of rumen epithelia.

The development of rumen microbiota in neonatal ruminants undergoes rapid and ongoing evolution and is susceptible to various environmental factors (33, 34). Previous research has indicated that the various early solid dietary regimes shaped the composition and structure of rumen microbiota in neonatal ruminants (35). In our study, we have demonstrated that the alfalfa hay introduction is advantageous for fostering the establishment of fiber-degrading microbiota in the rumen, which is largely due to the increase in fiber content, creating a suitable environment for the colonization of these fiber-degrading bacteria, such as *Succiniclasticum* (36), *Christensenellaceae* R-7 group (37, 38), *Prevotellaceae* UCG-001 (39), *Succinivibrio* (40), and *Lachnospira* (41). Concurrently, a robust correlation exists between the abundances of these significantly altered microbes and VFA levels. Notably, *Muribaculaceae_norank*, *Succiniclasticum*, and *Prevotellaceae* UCG-001 indicated the capacity for prolific propionate production, while *Muribaculaceae_norank* and *Mitsuokella* contributed to butyrate production (42, 43). These findings account for the primary changes observed in ruminal VFA levels. The modulation of these microbes and VFAs plays a pivotal role in regulating the rumen’s microecology, thereby fostering accelerated and improved growth in lambs. Concurrently, the addition of alfalfa hay induced modifications in the abundance of genes associated with acetate, propionate, and butyrate metabolism. Simultaneously, the addition of alfalfa hay has simplified the microbial network in the rumen contents. This phenomenon might be attributed to the heightened fiber content in alfalfa hay, which could steer the microbial community within the rumen contents toward fiber fermentation and degradation. Consequently, this shift leads to a reduction in the complexity of the microbial network. However, the precise mechanisms underlying this alteration remain to be fully elucidated.

The degradation of complex carbohydrates within the rumen is a coordinated process involving multiple enzymes, including AA, CBM, CE, GH, GT, PL, and SLH. Changes in the CAZy system serve as indicators of alterations in microbial functionality (44). In our experiment, we observed a notable decrease in the relative abundance of CBM and GHs enzyme genes in rumen content microbiota following alfalfa hay supplementation. This observation suggests that the introduction of alfalfa hay has a negative impact on the rumen microbiota’s capacity to degrade carbohydrates, which may potentially be attributed to the shifts in the abundance of ruminal microbes that secrete these specific enzymes (45). GHs represent a category of enzymes found in organisms, playing a pivotal role in the degradation and conversion of carbohydrates. Their primary function involves the hydrolysis of glycosidic bonds, leading to the breakdown of various glycosidic compounds into either monosaccharides or oligosaccharides. Wang et al.’s research (46) supports this observation, indicating that an increase in the proportion of dietary fiber leads to a decrease in the abundance of the GH family, particularly in the expression of families such as GH3. Families like GH2 and GH3, which are secreted by different microorganisms, primarily function as oligosaccharide-degrading enzymes (47). Moreover, CBMs play an indispensable role in the degradation of polysaccharides within organisms by enhancing enzyme binding, localization, and efficiency. This, in turn, facilitates the effective breakdown of polysaccharides. Different CBM types exhibit unique polysaccharide binding specificities, enabling them to operate effectively in diverse metabolic environments (48). Nevertheless, the introduction of alfalfa hay evidently diminishes the efficacy of CBMs. This indicates that the inclusion of alfalfa hay results in a decline in the enzymes' capacity within the rumen contents to bind with carbohydrates. The precise causes behind this decrease warrant further investigation. In addition, the abundances of AA3, CE4, and CE9 significantly increased, which is closely linked to rumen energy metabolism, ketone body production, and acid-base balance (49). Among these, CE9 exhibits a pronounced capacity for fiber breakdown, which may enhance rumen fermentation capability (50). In summary, the alterations in CAZy profiles may signify an augmentation in the population of fiber-degrading bacteria, leading to a shift in the relative proportions of various microbial species or subspecies within the rumen. As illustrated by the network correlations, it is evident that the complexity within the AH group has diminished. It is plausible that specific fiber-degrading bacteria may supplant the previously dominant producers of CBM and GH family enzymes.

Ruminal epithelial bacteria represent specific microbial ecological niches different from those associated with rumen contents, which function specific roles in recycling of epithelial tissue, scavenging of oxygen, and hydrolysis of urea (51). Our findings indicate that the inclusion of alfalfa hay increased the relative abundance of *Succiniclasticum*, *Megasphaera, Alloprevotella*, *Succinivibrio*, and *Suttonella* attached to rumen epithelia (52). *Succiniclasticum* (53) and *Succinivibrio* (40) are pivotal bacteria involved in the degradation of fiber and the production of succinic acid. They create conducive environments for the synthesis of propionic and butyric acids. Additionally, *Alloprevotella* is a substantial contributor to butyric acid production (54). Subsequent to the inclusion of alfalfa hay, the notable surge in the abundance of these microorganisms fosters the production of propionic and butyric acids, thereby contributing to the promotion of rumen epithelial cell development. The genus of *Succiniclasticum* has been demonstrated to enhance transcription and cell signaling pathway metabolism, consequently fostering the development of rumen epithelial cells (55, 56). *Alloprevotella* (54) exhibits antioxidant capabilities, while *Intestinimonas* (57) and *Dialister* (58) are regarded as pro-inflammatory microorganisms, given their propensity to induce an increased release of inflammatory mediators by immune cells. *Desulfovibrio* (59) possesses the ability to modulate the permeability of the animal’s intestinal tract and produce substances that are detrimental to the gut, consequently leading to intestinal damage. The decrease in the attachment of these potentially harmful microorganisms to the rumen epithelium plays a role in alleviating epithelial cell inflammation and facilitating the healthy development of epithelial cells.

In this study, lambs fed with alfalfa hay showed a significant downregulation of *DDX58* (60), *CXCL9* (61), and *SERPINE2* (62) in rumen tissue. This suggests a potential reduction in epithelial inflammation levels and an augmentation of immune capabilities. Furthermore, *FABP4* is associated with the PPAR signaling pathway, which also plays a role in regulating rumen epithelial fatty acid metabolism and oxidative stress (63). These genes encode proteins associated with immune responses and inflammation, signifying an augmentation in the immune capacity of the rumen epithelial tissue. These aspects should not be disregarded in the context of promoting rumen development. These findings align with the results of Yang et al.’s research (64), where alfalfa forage was introduced into lamb diets. The variations observed in genes associated with inflammation and immunity may also be interconnected with shifts in the microbiota adhering to the rumen epithelium (65), thereby providing additional support for the previously mentioned reduction in the attachment of potentially harmful bacteria.

Notably, the MEred module exhibited significant enrichment in pathways encompassing gluconeogenesis, the Notch signaling pathway, and positive regulation of epithelial cell proliferation. These pathways bear distinct relevance to the nutritional metabolism and cell development of the rumen epithelium, potentially serving as significant factors in driving rapid rumen epithelial development (66, 67). The Notch signaling pathway plays a crucial role in determining cell fate by influencing cell differentiation, proliferation, and apoptosis (67). It guides stem cells toward differentiation into specific cell types at different developmental stages and in various tissues, promoting tissue development (68). It also impacts T-cell development and differentiation, making it vital for the regulation of adaptive immune responses (67, 69). In the MEbrown module, cell division, epithelial cell differentiation, and positive regulation of innate immune response are prominently featured. This indicates that the introduction of alfalfa hay induces significant changes in the pathways related to epithelial cell proliferation and development (70, 71). This also explains the reasons behind the alterations in rumen papillae width and length. Concurrently, the microbial network adhering to the epithelium becomes more intricate, a phenomenon that proves beneficial for stimulating epithelial development and nutrient degradation (72, 73). Hence, it can be deduced that alfalfa hay has the capacity to modulate the immune status of the rumen epithelium, elevate the expression of pathways associated with rumen epithelial development, and consequently promote the development of the rumen epithelia.

### Conclusions

**Fig 6:**
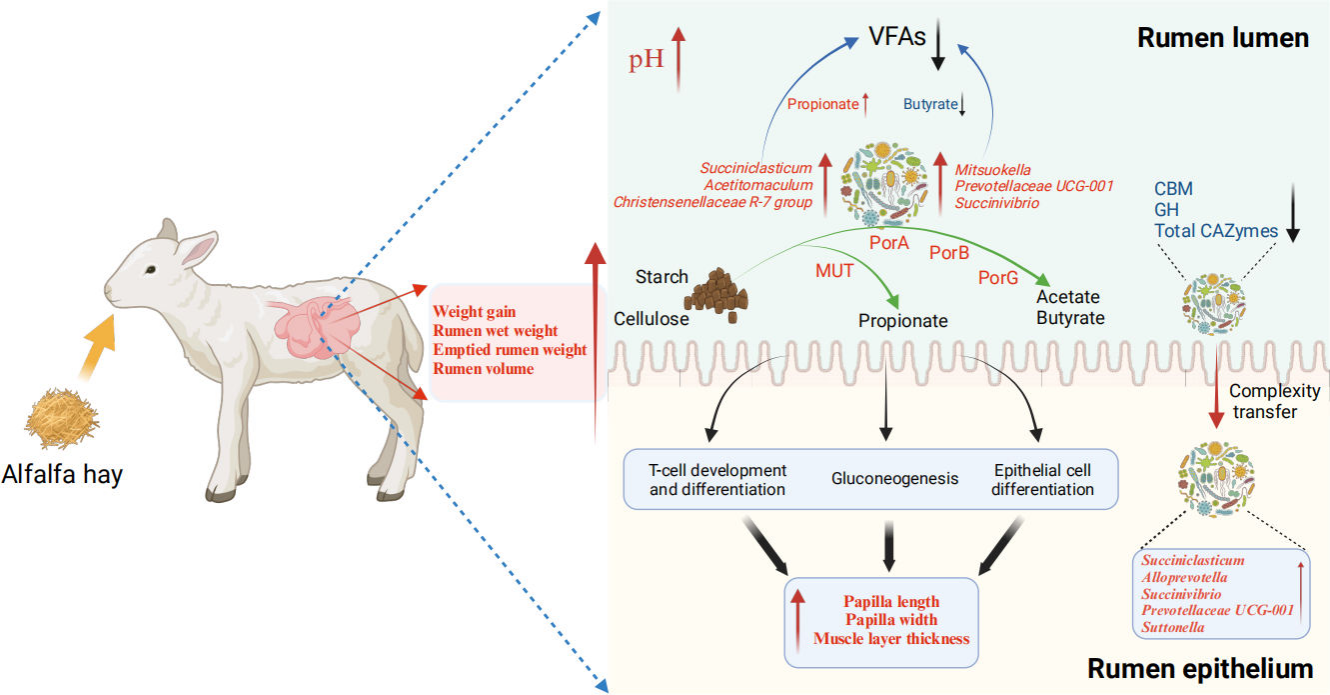
A summary of the results in the present study (created by Biorender.com).

This study has yielded novel insights into the role of alfalfa hay in fostering rumen epithelial development in young ruminants. The findings revealed that alfalfa hay was associated with increased length of rumen epithelial papillae and thickness of the muscle layer. Additionally, it stimulated propionate and butyrate metabolism, induced alterations in the abundance of carbohydrate enzymes within the rumen, influenced the expression of epithelial genes and metabolic pathways, and importantly, shifted the complexity of the rumen microbiota from the rumen contents to the rumen epithelia. These outcomes bear significant implications for the enhancement of rumen development (Fig. 6). Alfalfa hay, being a natural feed source, exhibits the capability to augment the developmental trajectory and efficiency of the rumen in young ruminants. This, in turn, exerts a favorable influence on the overall health and farming efficiency of lambs. By amalgamating methodologies like metagenomics and metabolomics, there exists an avenue for further exploration into potential interactions among high-fiber forage diet, rumen microbiota, and rumen epithelial development. Such investigations have the potential to advance the healthy and efficient rumen development by early nutritional interventions in young ruminants.

## Data Availability

The 16S rRNA sequencing data are available at the National Center for Biotechnology Information under accession numbers PRJNA1019655 and PRJNA881260; the metagenomic data analyzed in this study are available at the National Center for Biotechnology Information under accession number PRJNA1051932. The transcriptomic data sets analyzed in this study are available at National Center for Biotechnology Information under accession number PRJNA1064839.

## AFTER EPUB

*[This article was published on 5 January 2024 with an incorrect number in the Data Availability statement. The number was corrected in the current version, posted on 22 January 2024.]*

## SUPPLEMENTAL MATERIAL

Supplemental material msystems.01034-23-s0001.docx Tables S1 to S3 and Fig. S1 to S9.

ASM does not own the copyrights to Supplemental Material that may be linked to, or accessed through, an article. The authors have granted ASM a non-exclusive, world-wide license to publish the Supplemental Material files. Please contact the corresponding author directly for reuse.
