# Supplementary material for: Ruminal microbiota-host crosstalks promote ruminal epithelial development in neonatal lambs with alfalfa hay introduction
Source: mSystems. 2024 Jan 5;9(2):e01034-23. doi: 10.1128/msystems.01034-23 (PMC10878101; doi:10.1128/msystems.01034-23)
Supplement: Supplemental material — Tables S1 to S3 and Fig. S1 to S9. [file msystems.01034-23-s0001.docx]

**Table S1 Ingredient and chemical composition of the diet (dry matter basis)**

| **Item** | **CON** | **AH** |
| --- | --- | --- |
| **Ingredient, % DM** |  |  |
| Alfalfa hay | 0.00 | 100.00 |
| Corn | 56.00 | 0.00 |
| Soybean meal | 31.00 | 0.00 |
| Middling | 4.00 | 0.00 |
| Whey powder | 5.00 | 0.00 |
| Stone powder | 1.00 | 0.00 |
| Dicalcium phosphate | 1.50 | 0.00 |
| NaCl | 0.50 | 0.00 |
| Premix^1^ | 1.00 | 0.00 |
| **Nutrition level** |  |  |
| ME, MJ/kg DM | 12.67 | 8.03 |
| CP, % DM | 19.62 | 18.76 |
| EE, % DM | 3.68 | 2.26 |
| CF, % DM | 3.35 | 22.29 |
| NDF, % DM | 10.23 | 45.00 |
| ADF, % DM | 5.00 | 35.00 |
| Ash, % DM | 3.16 | 7.46 |
| Ca, % DM | 1.04 | 1.38 |
| P, % DM | 0.50 | 0.50 |

^1^ The premix was provided for the per kg diet: MnSO_4_ 153 mg, ZnSO_4_ 186 mg, FeSO_4_ 125 mg, CoCl_2_ 8.25 mg, KIO_3_ 25 mg, CuSO_4_ 33 mg, NaSeO_3_ 4 mg, VA 15.28 mg and VE 0.47 mg. CON: control group, AH: alfalfa hay group.

**Table S2 Description of the correlation between rumen contents and microbial networks in epithelium in CON group and AH group**

| id | degree | closenessCentrality | betweennessCentrality |
| --- | --- | --- | --- |
| Rumen content-CON group |  |  |  |
| Lachnospiraceae NK3A20 group | 17 | 26.91666667 | 131.8279572 |
| _Eubacterium_ nodatum group | 16 | 26.41666667 | 117.0015227 |
| Solobacterium | 16 | 26.41666667 | 117.0015227 |
| Sphingomonas | 16 | 28.16666667 | 686.2217166 |
| Olsenella | 15 | 25.91666667 | 66.56687419 |
| _Ruminococcus_ gauvreauii group | 15 | 25.91666667 | 66.56687419 |
| Lachnospira | 15 | 25.91666667 | 66.56687419 |
| Prevotella_7 | 15 | 25.75 | 475.1291707 |
| Phyllobacterium | 13 | 23.41666667 | 20.16543006 |
| Methanobrevibacter | 13 | 23.41666667 | 16.44328656 |
| Methanosphaera | 13 | 22.61666667 | 24.67120504 |
| Catenisphaera | 13 | 22.61666667 | 24.67120504 |
| Dialister | 12 | 24.5 | 65.29598725 |
| Family XIII AD3011 group | 12 | 22.61666667 | 40.97161955 |
| Megasphaera | 11 | 21.03333333 | 19.63333333 |
| Oribacterium | 11 | 20.7 | 6.433333333 |
| Prevotella | 11 | 20.7 | 6.433333333 |
| Pseudoramibacter | 11 | 21.61666667 | 7.567128778 |
| Erysipelotrichaceae UCG-002 | 11 | 21.61666667 | 7.567128778 |
| Erysipelotrichaceae UCG-009 | 11 | 21.61666667 | 7.567128778 |
| Oscillospiraceae_uncultured | 11 | 22.11666667 | 12.61584074 |
| Selenomonas | 10 | 20.36666667 | 6.066666667 |
| Bacteroidales_norank | 10 | 20.03333333 | 2 |
| Prevotellaceae_Unclassified | 10 | 20.03333333 | 2 |
| Acidaminococcus | 10 | 20.36666667 | 6.066666667 |
| Prevotella_9 | 10 | 20.36666667 | 6.066666667 |
| Selenomonadaceae_uncultured | 9 | 19.53333333 | 0 |
| Syntrophococcus | 9 | 21.83333333 | 20.72595171 |
| Aliihoeflea | 8 | 22.08333333 | 57.26562519 |
| Halomonas | 8 | 20.41666667 | 2.227380952 |
| Muribaculaceae_norank | 8 | 21.25 | 14.34913695 |
| Bifidobacterium | 8 | 21.25 | 14.34913695 |
| _Eubacterium_ coprostanoligenes group_norank | 7 | 19.45 | 9.559861535 |
| Mitsuokella | 7 | 21.75 | 95.07944178 |
| Allisonella | 7 | 19.45 | 99.68333333 |
| Acetitomaculum | 6 | 21.08333333 | 43.21895309 |
| Pseudoscardovia | 5 | 20.58333333 | 11.6 |
| Chloroplast_norank | 5 | 17.53333333 | 0 |
| Shuttleworthia | 5 | 18.11666667 | 0 |
| Clostridia UCG-014_norank | 5 | 20.58333333 | 76.4227055 |
| Bradyrhizobium | 5 | 5 | 0 |
| Allorhizobium-Neorhizobium-Pararhizobium-Rhizobium | 5 | 5 | 0 |
| Acinetobacter | 5 | 5 | 0 |
| Paracoccus | 5 | 5 | 0 |
| Aureimonas | 5 | 5 | 0 |
| Roseomonas | 5 | 5 | 0 |
| Rumen content-AH group |  |  |  |
| Ruminococcus | 12 | 23.28333333 | 97.05488598 |
| Prevotella | 12 | 23.71666667 | 153.6773157 |
| Mitsuokella | 11 | 23.75 | 202.5253183 |
| Oribacterium | 11 | 23.75 | 202.5253183 |
| Roseburia | 9 | 23.35 | 177.8745221 |
| _Ruminococcus_ gauvreauii group | 9 | 23.35 | 177.8745221 |
| Dialister | 9 | 21.45 | 81.29560721 |
| Selenomonadaceae_uncultured | 9 | 21.45 | 81.29560721 |
| _Eubacterium_ ruminantium group | 9 | 21.53333333 | 28.03278483 |
| Anaerovibrio | 9 | 21.53333333 | 28.03278483 |
| _Eubacterium_ eligens group | 9 | 21.53333333 | 28.03278483 |
| Schwartzia | 9 | 21.53333333 | 28.03278483 |
| Megasphaera | 7 | 21.28333333 | 93.46668855 |
| Lachnospiraceae NK3A20 group | 7 | 21.28333333 | 93.46668855 |
| Methanobrevibacter | 7 | 20.81666667 | 202.1441858 |
| Solobacterium | 6 | 20.61666667 | 462.0784468 |
| NK4A214 group | 6 | 19.11666667 | 164.444925 |
| Selenomonas | 6 | 20.63333333 | 271.4845726 |
| Erysipelotrichaceae UCG-002 | 6 | 20.65 | 355.2246315 |
| Suttonella | 6 | 19.38333333 | 158.1100717 |
| Lachnospira | 5 | 18.41666667 | 254.1272896 |
| _Eubacterium_ nodatum group | 5 | 19.71666667 | 126.1480608 |
| Acetitomaculum | 5 | 19.86666667 | 343.1000136 |
| Olsenella | 5 | 17.83333333 | 153.5668926 |
| Sharpea | 5 | 19.35 | 91.22104635 |
| _Eubacterium_ coprostanoligenes group_norank | 5 | 19.05952381 | 130.6289629 |
| Pseudobutyrivibrio | 4 | 19.63333333 | 241.9266234 |
| Bacteroidales_norank | 4 | 15.96666667 | 126.4839371 |
| Pseudoscardovia | 4 | 16.05 | 184 |
| Acidaminococcus | 4 | 17.95 | 0 |
| Alloprevotella | 4 | 17.95 | 0 |
| Syntrophococcus | 4 | 18.01666667 | 78.66482684 |
| Methanosphaera | 4 | 18.9 | 71.34311186 |
| Christensenellaceae R-7 group | 4 | 18.55952381 | 36.62896295 |
| Desulfovibrio | 4 | 18.35 | 165.0449761 |
| Prevotella_7 | 3 | 16.05 | 0 |
| Chloroplast_norank | 3 | 16.05 | 0 |
| Lachnospiraceae FE2018 group | 3 | 17.41666667 | 101.4722146 |
| Succinivibrio | 3 | 14.82619048 | 114.0884083 |
| Atopobiaceae_uncultured | 3 | 13.8547619 | 55.72360293 |
| Prevotellaceae UCG-001 | 3 | 17.03333333 | 94 |
| Catenisphaera | 2 | 12.23928571 | 0 |
| Clostridia UCG-014_norank | 2 | 12.31904762 | 0 |
| Succiniclasticum | 2 | 12.31904762 | 0 |
| Prevotellaceae_Unclassified | 2 | 13.89285714 | 0 |
| F082_norank | 2 | 14.71904762 | 89.1566232 |
| Muribaculaceae_norank | 1 | 11.72857143 | 0 |
| Rikenellaceae RC9 gut group | 1 | 12.51666667 | 0 |
| Shuttleworthia | 1 | 13.37738095 | 0 |
| Epithelial-CON group |  |  |  |
| Oribacterium | 8 | 17.57857143 | 196.3712121 |
| Campylobacter | 7 | 17.56666667 | 350.8237374 |
| Thauera | 7 | 17.21190476 | 237.1515152 |
| Rikenellaceae RC9 gut group | 7 | 15.4202381 | 35.19444444 |
| Clostridia UCG-014_norank | 7 | 15.4202381 | 35.19444444 |
| Alloprevotella | 7 | 15.4202381 | 16.69444444 |
| Cloacibacillus | 6 | 17.85 | 464.8323232 |
| Intestinimonas | 6 | 15.84285714 | 91.9540404 |
| Prevotella | 6 | 14.9202381 | 24.5 |
| Prevotella_7 | 5 | 14.39404762 | 117.3333333 |
| Dialister | 5 | 16.15952381 | 528 |
| _Ruminococcus_ gauvreauii group | 5 | 14.39404762 | 117.3333333 |
| Staphylococcus | 5 | 12.57063492 | 156 |
| Succiniclasticum | 5 | 14.39404762 | 117.3333333 |
| Oscillospiraceae_uncultured | 5 | 15.05952381 | 86.04393939 |
| Succinivibrio | 5 | 14.95 | 42.14444444 |
| Parabacteroides | 5 | 16.71666667 | 304.1323232 |
| Mitsuokella | 4 | 11.90396825 | 80 |
| _Eubacterium_ nodatum group | 4 | 13.77857143 | 152.1444444 |
| Family XIII AD3011 group | 4 | 14.96190476 | 253.1777778 |
| Prevotellaceae UCG-001 | 4 | 16.65 | 512.8313131 |
| Butyrivibrio | 4 | 16.55 | 506.220202 |
| Lachnospiraceae NK3A20 group | 4 | 13.94285714 | 25.58030303 |
| Muribaculaceae_norank | 4 | 12.96309524 | 1.833333333 |
| Bacteroides | 4 | 14.3452381 | 53.84949495 |
| Syntrophococcus | 3 | 12.62738095 | 156 |
| Desulfovibrio | 3 | 13.50952381 | 110.5242424 |
| Erysipelotrichaceae UCG-002 | 3 | 13.99285714 | 168.1333333 |
| Nitrosospira | 3 | 13.20238095 | 80 |
| Howardella | 3 | 11.54880952 | 0 |
| _Eubacterium_ coprostanoligenes group_norank | 2 | 10.1547619 | 80 |
| Selenomonadaceae_uncultured | 2 | 2 | 2 |
| Pseudoscardovia | 2 | 13.63333333 | 0 |
| Shuttleworthia | 2 | 13.66190476 | 112.5242424 |
| Ruminococcus | 2 | 12.71666667 | 146.1444444 |
| Acetitomaculum | 2 | 9.605555556 | 0 |
| Solobacterium | 2 | 9.605555556 | 0 |
| F082_norank | 2 | 11.71071429 | 0 |
| Roseburia | 2 | 12.2952381 | 80 |
| Unclassified | 1 | 1.5 | 0 |
| Olsenella | 1 | 8.091269841 | 0 |
| Desulfobulbus | 1 | 9.816666667 | 0 |
| Asteroleplasma | 1 | 8.855555556 | 0 |
| Lachnospiraceae FE2018 group | 1 | 1.5 | 0 |
| Lachnospiraceae UCG-008 | 1 | 9.463095238 | 0 |
| Epithelial-AH group |  |  |  |
| Suttonella | 8 | 19.09285714 | 371.199253 |
| Oscillospiraceae_uncultured | 8 | 18.70952381 | 165.8836601 |
| Olsenella | 8 | 16.45238095 | 115.3333333 |
| Clostridia UCG-014_norank | 7 | 18.55 | 184.5923436 |
| _Eubacterium_ coprostanoligenes group_norank | 7 | 18.55 | 184.5923436 |
| Rikenellaceae RC9 gut group | 7 | 16.63452381 | 93.45294118 |
| Solobacterium | 7 | 17.92619048 | 190.3563025 |
| Selenomonadaceae_uncultured | 7 | 18 | 595.3333333 |
| Bacteroides | 6 | 17.00952381 | 97.13333333 |
| Ruminococcus | 6 | 17.34285714 | 203.6590103 |
| Lachnospiraceae NK3A20 group | 6 | 17.70952381 | 74.69112979 |
| Nitrosospira | 6 | 15.80119048 | 63.84705882 |
| _Ruminococcus_ gauvreauii group | 6 | 17.70952381 | 74.69112979 |
| Roseburia | 6 | 15.45238095 | 70 |
| Shuttleworthia | 6 | 15.45238095 | 70 |
| Campylobacter | 5 | 16.06666667 | 401.9409897 |
| Acidaminococcus | 5 | 17.85 | 525.8521008 |
| Prevotella | 5 | 13.29761905 | 4 |
| _Eubacterium_ ruminantium group | 5 | 13.29761905 | 82 |
| Megasphaera | 5 | 14.78571429 | 1.333333333 |
| Alloprevotella | 5 | 16.59285714 | 77.85555556 |
| Mitsuokella | 4 | 13.2452381 | 161 |
| Prevotellaceae UCG-001 | 4 | 15.37619048 | 0 |
| Howardella | 4 | 14.42619048 | 142.636788 |
| Erysipelotrichaceae UCG-002 | 4 | 14.30357143 | 61.33333333 |
| Dialister | 4 | 14.28571429 | 35 |
| Blvii28 wastewater-sludge group | 4 | 16.46666667 | 108.2444444 |
| Lachnoclostridium | 4 | 17.4 | 476.8674136 |
| Staphylococcus | 4 | 17.33333333 | 307.7563025 |
| Oribacterium | 3 | 13.17857143 | 18 |
| Succinivibrio | 3 | 14.55357143 | 115.0145658 |
| Acetitomaculum | 3 | 12.13095238 | 1 |
| Muribaculaceae_norank | 3 | 12.36706349 | 0 |
| Lachnospiraceae FE2018 group | 3 | 12.36706349 | 0 |
| Desulfovibrio | 3 | 13.39642857 | 33.4 |
| Lachnospiraceae UCG-008 | 3 | 14.13452381 | 0 |
| Family XIII AD3011 group | 3 | 14.13452381 | 0 |
| Prevotella_7 | 2 | 12.07857143 | 0 |
| Thauera | 2 | 10.44166667 | 82 |
| Xanthomonas | 2 | 11.14325397 | 0 |
| Unclassified | 1 | 8.289285714 | 0 |
| Asteroleplasma | 1 | 10.48928571 | 0 |
| Pseudoscardovia | 1 | 1 | 0 |
| Syntrophococcus | 1 | 1 | 0 |
| Succiniclasticum | 1 | 9.575793651 | 0 |
| _Eubacterium_ nodatum group | 1 | 1 | 0 |
| F082_norank | 1 | 1 | 0 |

**Table S3 Top 30 biological process terms significantly enriched in the MEred and Mbrown module.**

| Term | Count | *P* Value | Genes |
| --- | --- | --- | --- |
| MEred |  |  |  |
| GO:0018057~peptidyl-lysine oxidation | 3 | 0.0017 | LOX, LOXL3, LOXL2 |
| GO:0045944~positive regulation of transcription from RNA polymerase II promoter | 14 | 0.0080 | BCAS3, HDAC2, CALCOCO1, SETD3, AKAP8L, BMP5, NFATC4, PLAC8, IFT74, CREB3L3, MYC, HES1, PAXBP1, ZNF410 |
| GO:0006919~activation of cysteine-type endopeptidase activity involved in apoptotic process | 4 | 0.0132 | EGLN3, MYC, F2R, HIP1R |
| GO:0006094~gluconeogenesis | 3 | 0.0180 | RBP4, TPI1, ATF4 |
| GO:0007507~heart development | 5 | 0.0214 | IFT74, MAP2K1, LOX, CC2D2A, NFATC4 |
| GO:0060412~ventricular septum morphogenesis | 3 | 0.0250 | WNT11, SLIT3, HES1 |
| GO:0060675~ureteric bud morphogenesis | 2 | 0.0259 | WNT11, HES1 |
| GO:0001837~epithelial to mesenchymal transition | 3 | 0.0302 | WNT11, LOXL3, LOXL2 |
| GO:0030178~negative regulation of Wnt signaling pathway | 3 | 0.0358 | APCDD1, DKK3, NFATC4 |
| GO:0060754~positive regulation of mast cell chemotaxis | 2 | 0.0387 | SWAP70, VEGFC |
| GO:0007155~cell adhesion | 6 | 0.0394 | LAMA2, COL5A1, MXRA8, CERCAM, HES1, ATP1B1 |
| GO:0001666~response to hypoxia | 4 | 0.0511 | EGLN3, MMP2, PLAT, SLC2A8 |
| GO:1904491~protein localization to ciliary transition zone | 2 | 0.0512 | TCTN1, CC2D2A |
| GO:0030308~negative regulation of cell growth | 4 | 0.0571 | WNT11, HYAL1, SPHK2, SLIT3 |
| GO:0034644~cellular response to UV | 3 | 0.0581 | MYC, BAK1, NFATC4 |
| GO:0050821~protein stabilization | 5 | 0.0621 | LAMP1, HIP1R, AHSP, ZSWIM7, ATP1B1 |
| GO:0019800~peptide cross-linking via chondroitin 4-sulfate glycosaminoglycan | 2 | 0.0636 | EGFLAM, NDNF |
| GO:0030282~bone mineralization | 3 | 0.0651 | WNT11, LOX, ATF4 |
| GO:0030199~collagen fibril organization | 3 | 0.0687 | COL5A1, LOX, LOXL2 |
| GO:0070301~cellular response to hydrogen peroxide | 3 | 0.0687 | PDGFRB, TRPA1, ZNF277 |
| GO:0006915~apoptotic process | 5 | 0.0721 | EGLN3, HIP1R, MYD88, MCL1, TNFRSF1A |
| GO:0050919~negative chemotaxis | 2 | 0.0758 | SLIT3, PLXNA3 |
| GO:0034316~negative regulation of Arp2/3 complex-mediated actin nucleation | 2 | 0.0758 | GMFG, HIP1R |
| GO:0007219~Notch signaling pathway | 4 | 0.0819 | CDH6, IFT74, GOT1, HES1 |
| GO:0050679~positive regulation of epithelial cell proliferation | 3 | 0.0839 | HYAL1, MYC, BMP5 |
| GO:0048538~thymus development | 3 | 0.0839 | CCNB2, MAP2K1, HES1 |
| GO:0090037~positive regulation of protein kinase C signaling | 2 | 0.0879 | WNT11, SPHK2 |
| GO:0070828~heterochromatin organization | 2 | 0.0879 | HDAC2, LOXL2 |
| GO:0071467~cellular response to pH | 2 | 0.0879 | HYAL1, MCOLN1 |
| GO:0021537~telencephalon development | 2 | 0.0879 | TCTN1, HES1 |
| MEbrown |  |  |  |
| GO:0031297~replication fork processing | 7 | 0.0005 | ZRANB3, GEN1, CDK9, BLM, PCNA, EME1, PRIMPOL |
| GO:0090267~positive regulation of mitotic cell cycle spindle assembly checkpoint | 4 | 0.0026 | GEN1, PCID2, NDC80, MAD2L1 |
| GO:0043330~response to exogenous dsRNA | 5 | 0.0027 | NFKBIA, KCNJ8, DDX1, CARD9, MAPK3 |
| GO:0006264~mitochondrial DNA replication | 4 | 0.0059 | MGME1, RRM1, POLG2, PRIMPOL |
| GO:0051660~establishment of centrosome localization | 3 | 0.0110 | FHOD1, CEP83, MAD2L1 |
| GO:0051301~cell division | 13 | 0.0111 | BORA, SPAG5, DYNLT3, NDC80, PPP1CA, CDC42, BABAM2, TPX2, CDK1, MAPRE3, SPDL1, ZNF830, LZTS2 |
| GO:0018279~protein N-linked glycosylation via asparagine | 4 | 0.0140 | ST6GAL1, ALG8, TUSC3, MAGT1 |
| GO:0006636~unsaturated fatty acid biosynthetic process | 4 | 0.0140 | FADS3, ELOVL4, SCD, FADS1 |
| GO:0007049~cell cycle | 11 | 0.0224 | BABAM2, TPX2, BORA, MCM7, DYNLT3, MCM3, MAPRE3, MCM6, ZNF830, LZTS2, SKA3 |
| GO:0045910~negative regulation of DNA recombination | 3 | 0.0260 | ZRANB3, BLM, MSH2 |
| GO:0032259~methylation | 8 | 0.0280 | TPMT, THUMPD3, DPH5, TYMS, METTL26, LCMT1, METTL15, GNMT |
| GO:0031642~negative regulation of myelination | 3 | 0.0354 | FIG4, KLK8, CTSC |
| GO:0031647~regulation of protein stability | 6 | 0.0367 | SREBF1, HSPA8, HDAC3, SUMO1, LMNA, LSS |
| GO:0048536~spleen development | 5 | 0.0374 | PCID2, PPP2R3C, BCL3, PKN1, NKX2-3 |
| GO:0030855~epithelial cell differentiation | 6 | 0.0401 | GSTK1, ACADVL, PCNA, ELF3, PGK1, CDK1 |
| GO:2001234~negative regulation of apoptotic signaling pathway | 4 | 0.0492 | ING2, QARS1, PCGF2, MEIS3 |
| GO:0006695~cholesterol biosynthetic process | 4 | 0.0492 | HMGCS1, HMGCS2, HMGCR, LSS |
| GO:0065003~macromolecular complex assembly | 5 | 0.0615 | TFAP4, GPAA1, NUPR1, CD247, JCHAIN |
| GO:0034501~protein localization to kinetochore | 3 | 0.0694 | IK, CDK1, SPDL1 |
| GO:0051290~protein heterotetramerization | 3 | 0.0694 | RRM1, KRT1, FARSB |
| GO:0036297~interstrand cross-link repair | 4 | 0.0710 | FANCL, FANCC, FANCG, FAN1 |
| GO:0006325~chromatin organization | 9 | 0.0784 | PRMT6, BABAM2, ING2, HDAC3, KDM1A, MSL3, DEK, ING1, TDRD3 |
| GO:0007052~mitotic spindle organization | 4 | 0.0791 | INTS13, DCTN2, RCC1, NDC80 |
| GO:0031663~lipopolysaccharide-mediated signaling pathway | 4 | 0.0791 | NFKBIA, PLCG2, CD14, MAPK3 |
| GO:0045089~positive regulation of innate immune response | 3 | 0.0824 | PLSCR1, POLR3B, CARD9 |
| GO:1900264~positive regulation of DNA-directed DNA polymerase activity | 3 | 0.0824 | PCNA, POLG2, DSCC1 |
| GO:1902600~hydrogen ion transmembrane transport | 3 | 0.0824 | RNASEK, ATP6AP1, ATP6V1B1 |
| GO:0010165~response to X-ray | 3 | 0.0824 | BLM, MSH2, XRCC2 |
| GO:0010142~farnesyl diphosphate biosynthetic process, mevalonate pathway | 2 | 0.0865 | HMGCS1, HMGCS2 |
| GO:0034472~snRNA 3'-end processing | 2 | 0.0865 | TOE1, INTS8 |


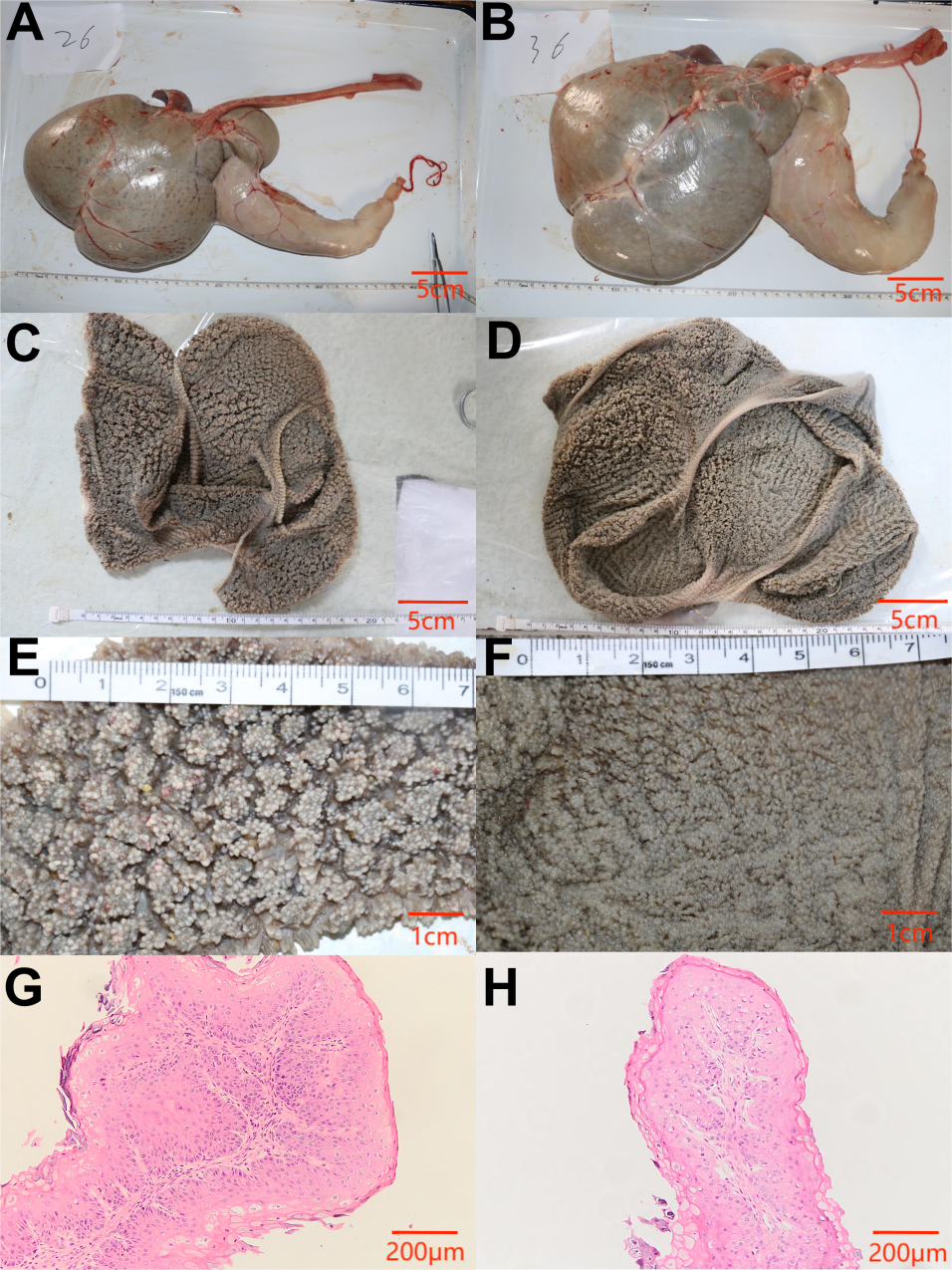
**Fig. S1 Rumen papilla morphology in lambs.** Among them, A, C and E are the rumen morphology of the CON group, B, D and H are the rumen morphology of the AH group, G and H are the rumen papilla of the CON group and the AH group observed under microscope, respectively.

**
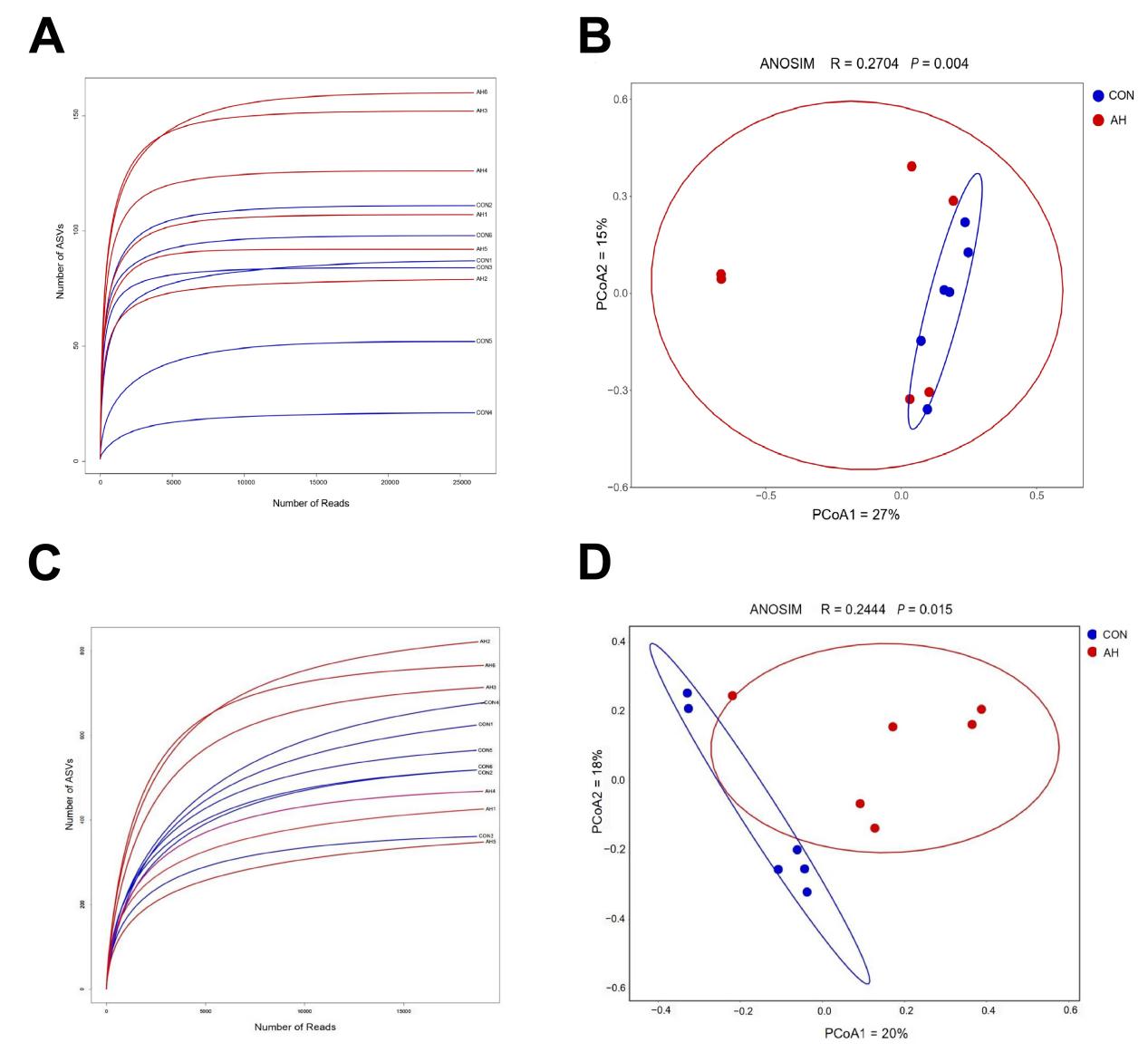
Fig. S2 Rumen contents and epithelium microbial of rarefaction curves and structural similarity.** Bacterial rarefaction curves of the bacterial communities of the rumen contents (A) and rumen epithelium (C), (Abscissa: the number of randomly selected sequencing data; ordinate: the number of ASV observed). Unweighted UniFrac principal coordinate analysis (PCoA) of the bacterial communities in the rumen contents (B) and rumen epithelium (D) between CON and AH. CON: control group, AH: alfalfa hay group.

**
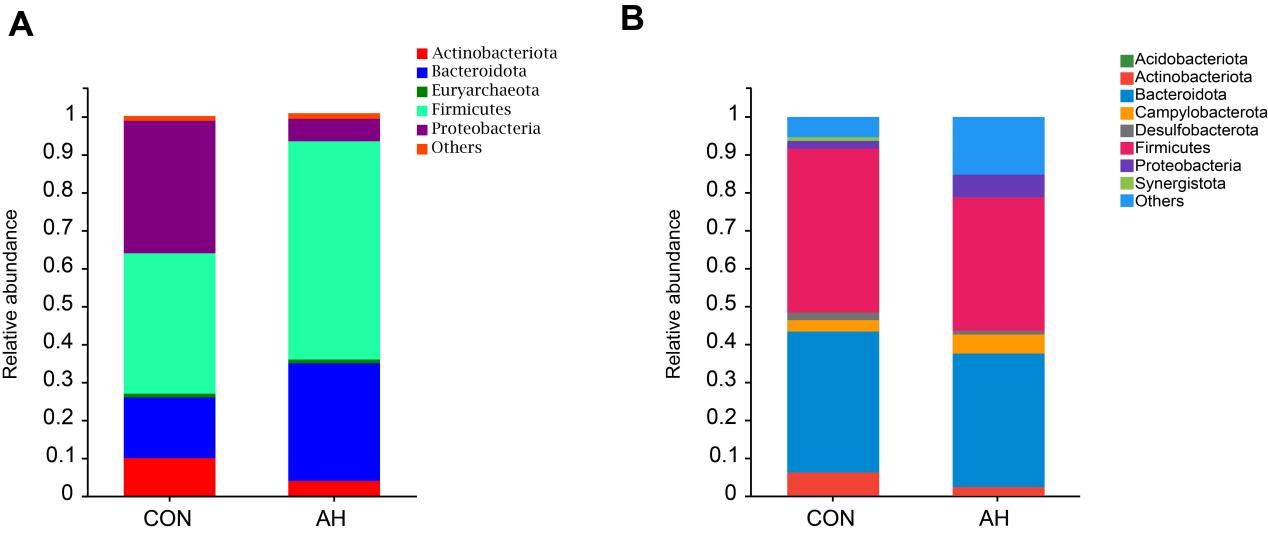
Fig. S3 Composition of bacterial phylum in rumen contents (A) and rumen epithelium (B).** CON: control group, AH: alfalfa hay group.

**
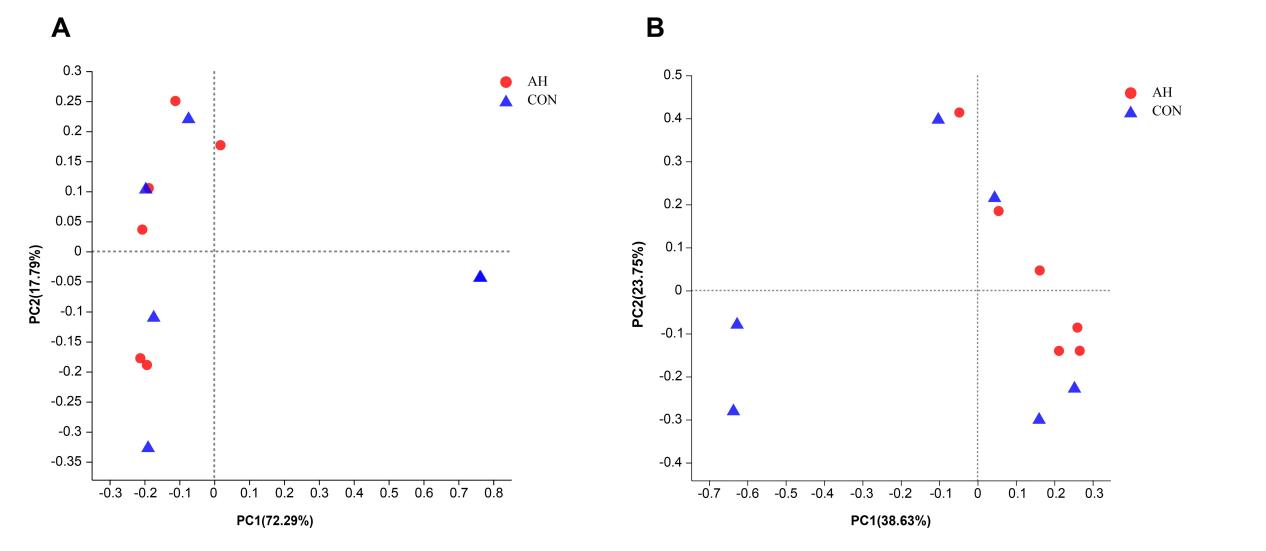
Fig. S4 PCoA polt at the bacterial phylum (A) and genus (B) level of the rumen contents.** CON: control group, AH: alfalfa hay group.

**
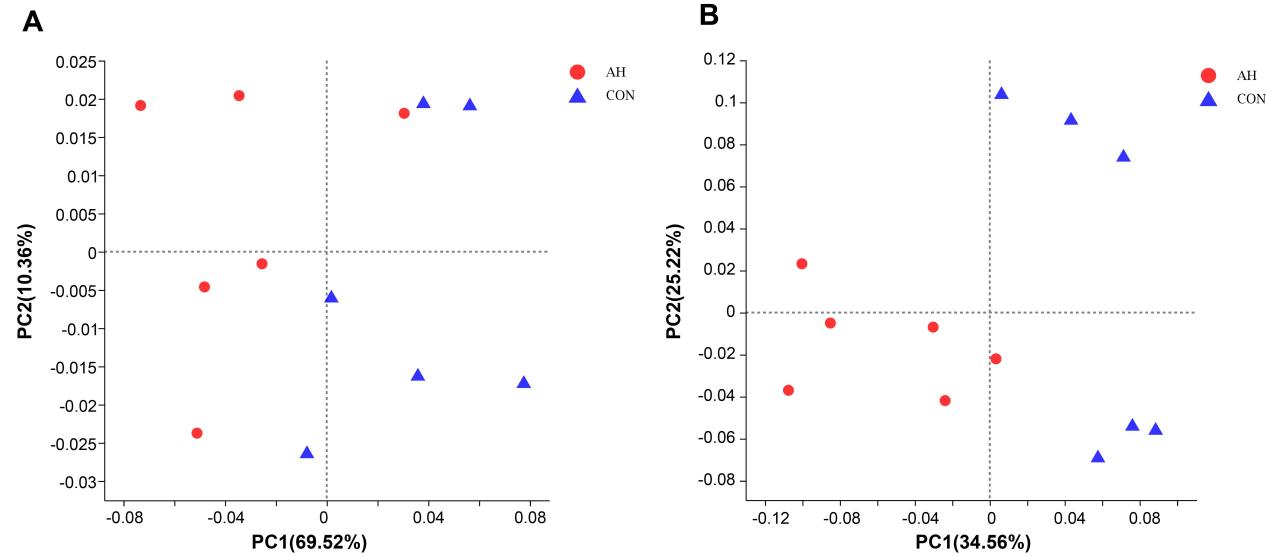
Fig. S5 PCoA plot at the class (A) and family(B) level in CAZys.** CON: control group, AH: alfalfa hay group.


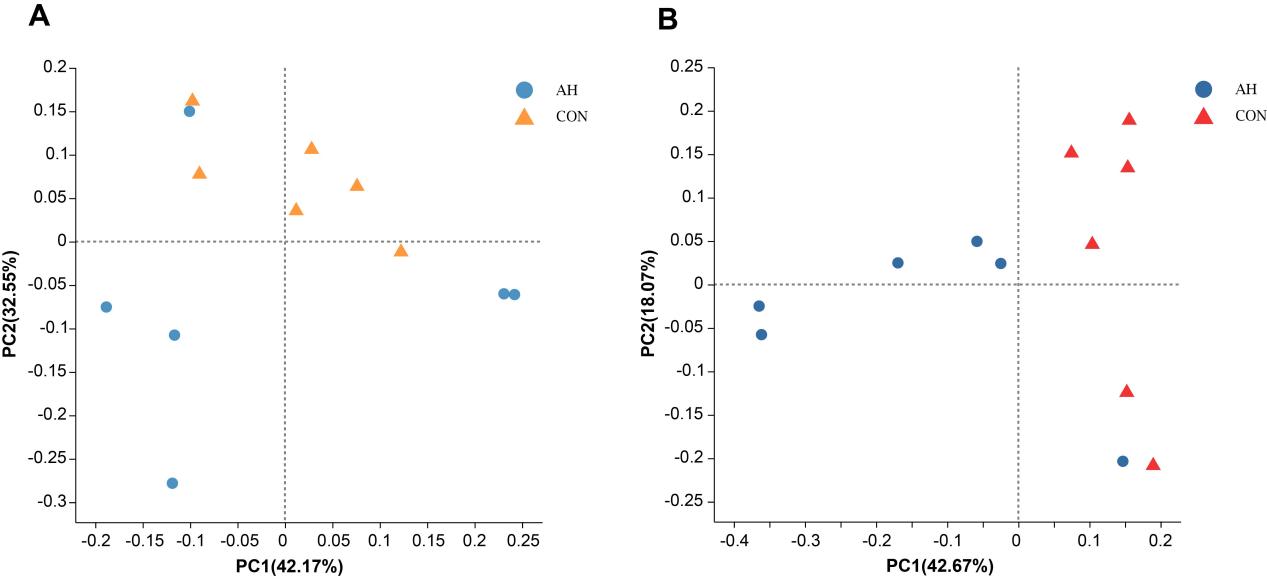
**Fig. S6 PCoA was conducted at the bacterial phylum (A) and genus (B) level of the rumen epithelium.**CON: control group, AH: alfalfa hay group.

**
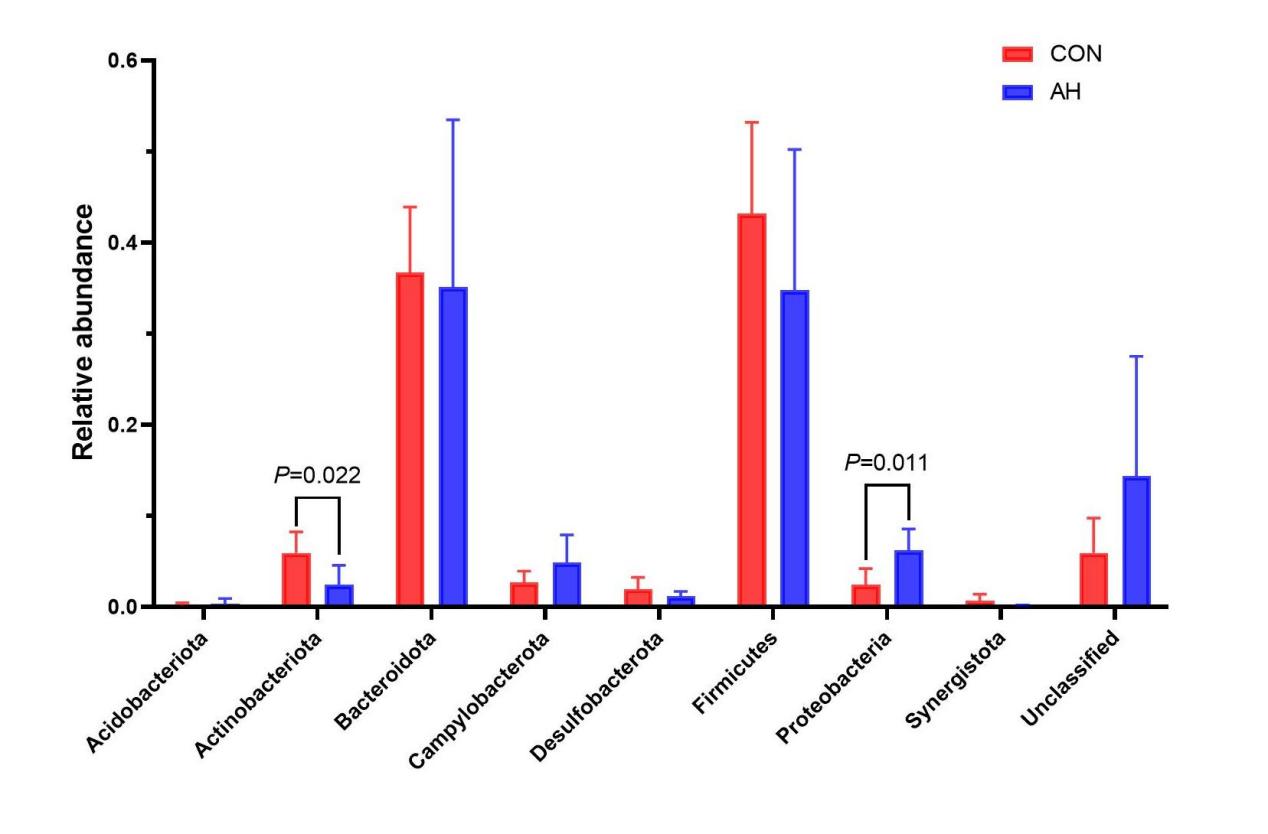
Fig. S7 Microbial phylum-level differences in the rumen epithelium.** CON: control group, AH: alfalfa hay group.

**
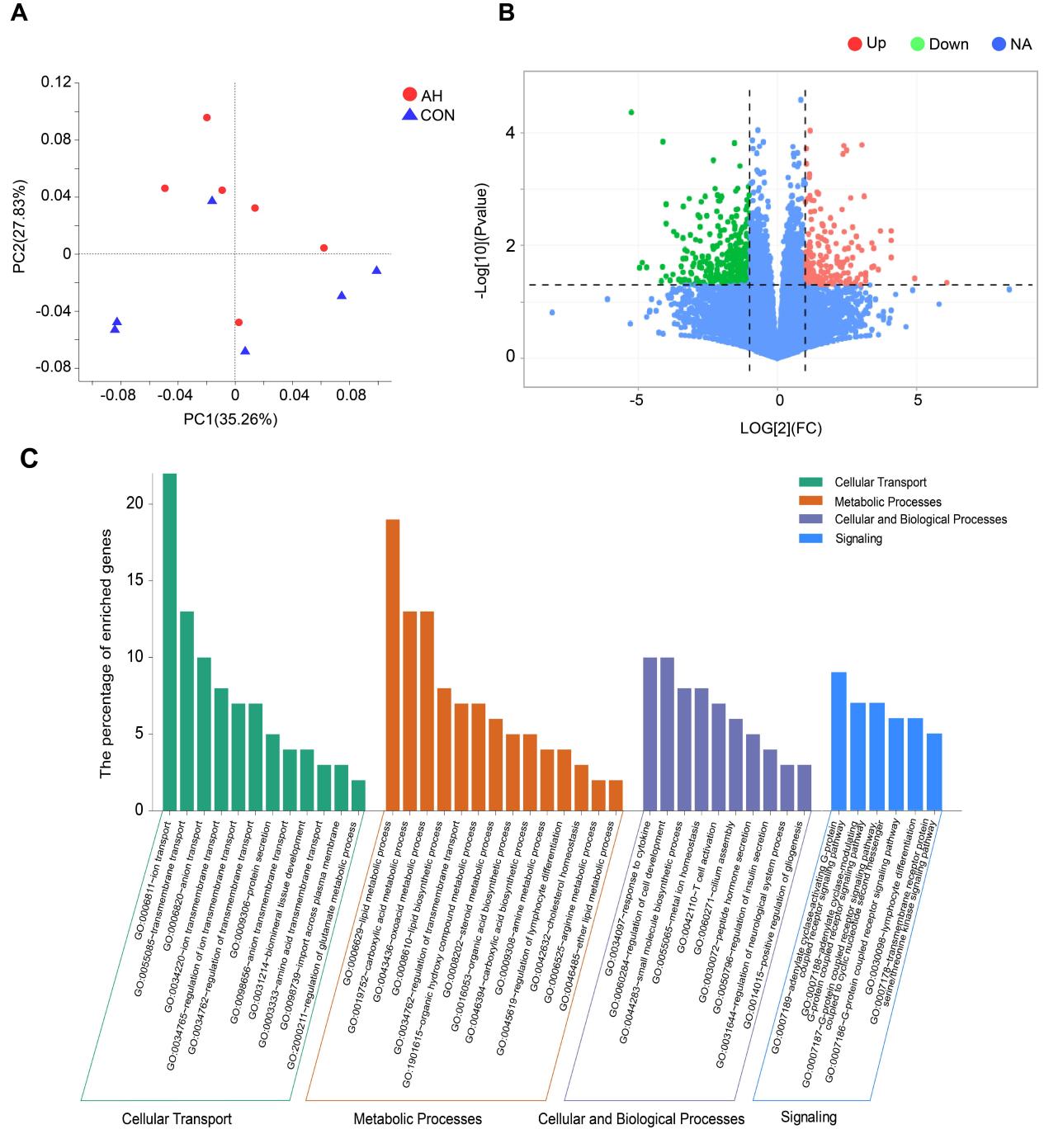
**

**Fig. S8 Transcriptome classification analysis. A: The compositional profiles rumen epithelial genes of based on PCoA. B: Differential expression analysis of rumen epithelial genes. C: Classification of GO Pathways with Significant Differences in Biological Processes into Cellular Transport, Metabolic Processes, Cellular and Biological Processes, and Signaling.** CON: control group, AH: alfalfa hay group.

**
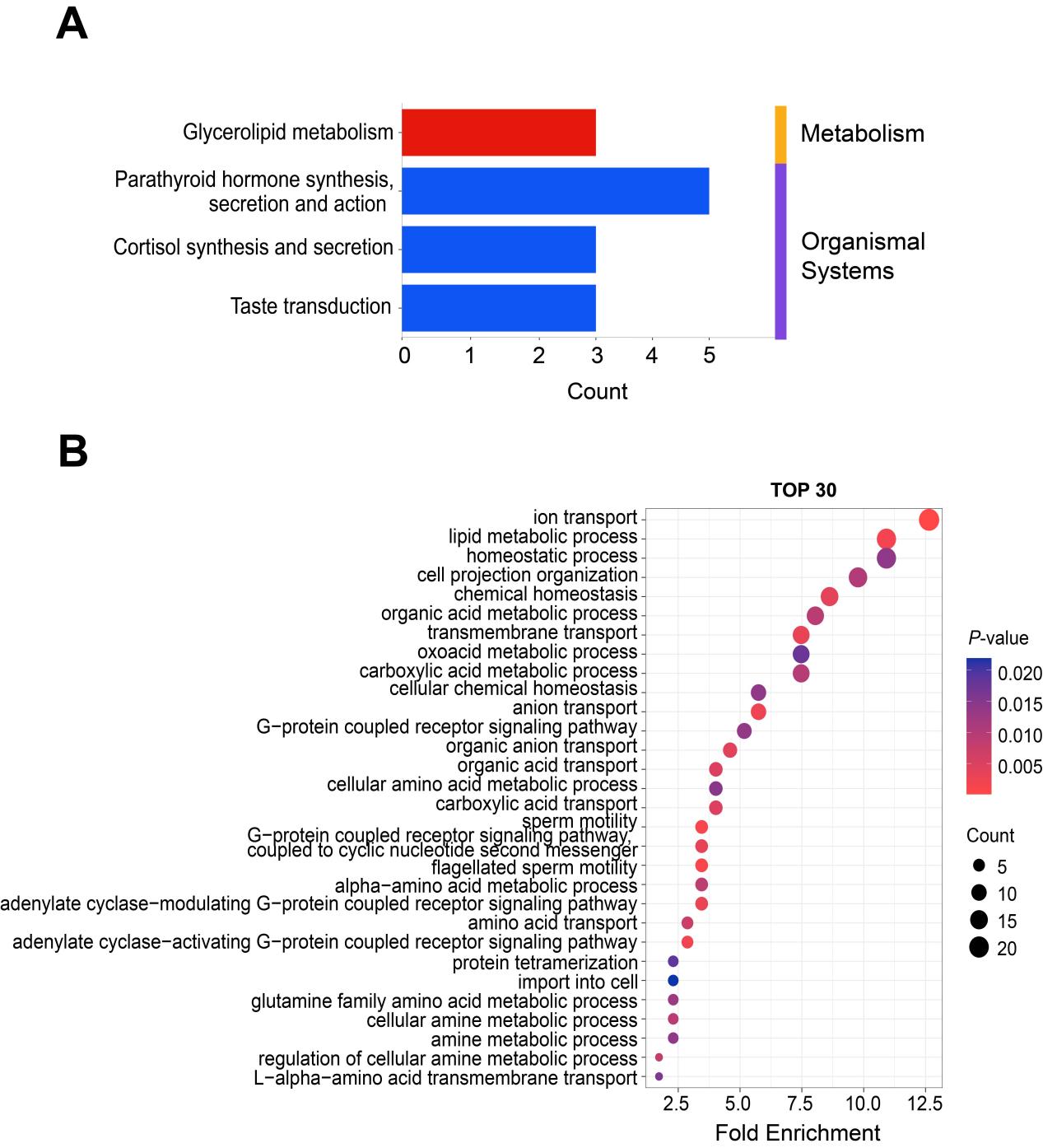
Fig. S9 Pathway enrichment analysis based on all DEGs**. A: KEGG pathway analysis. B: GO enrichment analysis of significantly difference genes in Biological Process (Top 30).
